# Recent Advances in Layered Double Hydroxide-Based Electrochemical and Optical Sensors

**DOI:** 10.3390/nano11112809

**Published:** 2021-10-22

**Authors:** Andrew Kim, Imre Varga, Arindam Adhikari, Rajkumar Patel

**Affiliations:** 1 Department of Chemical Engineering, The Cooper Union for the Advancement of Science and Art, New York, NY 10003, USA; kim70@cooper.edu; 2 Institute of Chemistry, Eötvös Loránd University, 1117 Budapest, Hungary; 3 Aadarsh Innovations, Pune 411045, India; arindam.adhikari@gmail.com; 4 Energy and Environmental Science and Engineering (EESE), Integrated Science and Engineering Division (ISED), Underwood International College, Yonsei University, 85 Songdogwahak-ro, Yeonsu-gu, Incheon 21983, Korea

**Keywords:** LDH, electrochemical sensors, optical sensors, reduced graphene oxide

## Abstract

Layered double hydroxides (LDHs) have attracted considerable attention as promising materials for electrochemical and optical sensors owing to their excellent catalytic properties, facile synthesis strategies, highly tunable morphology, and versatile hosting ability. LDH-based electrochemical sensors are affordable alternatives to traditional precious-metal-based sensors, as LDHs can be synthesized from abundant inorganic precursors. LDH-modified probes can directly catalyze or host catalytic compounds that facilitate analyte redox reactions, detected as changes in the probe’s current, voltage, or resistance. The porous and lamellar structure of LDHs allows rapid analyte diffusion and abundant active sites for enhanced sensor sensitivity. LDHs can be composed of conductive materials such as reduced graphene oxide (rGO) or metal nanoparticles for improved catalytic activity and analyte selectivity. As optical sensors, LDHs provide a spacious, stable structure for synergistic guest–host interactions. LDHs can immobilize fluorophores, chemiluminescence reactants, and other spectroscopically active materials to reduce the aggregation and dissolution of the embedded sensor molecules, yielding enhanced optical responses and increased probe reusability. This review discusses standard LDH synthesis methods and overviews the different electrochemical and optical analysis techniques. Furthermore, the designs and modifications of exemplary LDHs and LDH composite materials are analyzed, focusing on the analytical performance of LDH-based sensors for key biomarkers and pollutants, including glucose, dopamine (DA), H_2_O_2_, metal ions, nitrogen-based toxins, and other organic compounds.

## 1. Introduction

Affordable, accurate, and rapid sensing technology is essential for monitoring the environment, controlling water quality, and diagnosing medical conditions. Global industrialization and urbanization have increased inorganic and organic pollution, threatening human health, other living organisms, and the environment [1,2,3]. Monitoring specific pollutants from complex environmental mixtures requires sensors with high selectivity and low limits of detection. Moreover, sensors with a fast response are critical for clinical diagnostics, such as measuring DA levels for Parkinson’s disease [4,5] or measuring glucose concentrations for diabetes [6]. Of the various analytical methods, the electrochemical and optical analytical techniques have received much attention, owing to their high selectivity, fast response, and portability [7,8,9]. Electrochemical sensors monitor the changes in electrical current caused by redox reactions between the target analyte and reusable probe, allowing for accurate, real-time, automatable, and reusable detection [10,11,12]. Optical sensors measure changes in the fluorescence or color of probes, enabling immediate “naked-eye” detection for harmful substances [13,14]. Much research has focused on designing LDH-based electrochemical and optical sensors.

LDHs refer to a family of lamellar nanomaterials comprising octahedral crystals with metal cation cores surrounded by charge-balancing hydroxides and anionic intercalants. The metal centers of LDHs can be monometallic with divalent cations but are often bimetallic with a mixture of divalent (M^2+^) and trivalent (M^3+^) cations. Such LDHs follow the general formula [M^2+^_1−x_ + M^3+^_x_ (OH)_2_]^x+^ (A^n−^)_x/n_·mH_2_O, where A^n−^ is the anionic intercalant [15]. Common divalent metals include Ni^2+^, Co^2+^, Mg^2+^; common trivalent metals include Al^3+^ and Fe^3+^; and common interlayer anions include CO_3_^2−^ and NO_3_^−^. LDH materials have large specific surface areas and exhibit high adsorption abilities for various substrates, owing to their sheet-like 2D structure. Thus, LDHs are versatile host materials with excellent guest–host synergies [16]. The anionic intercalants can be exchanged, allowing highly tunable LDH characteristics [17]. LDHs are stable nanostructures due to the strong electrostatic interactions and hydrogen bonding within and between the brucite-like layers [18]. Due to their advantageous characteristics, LDHs have been extensively used in catalysis [19,20,21,22], drug delivery [23,24,25,26], substrate extraction [27,28,29,30,31], pollutant degradation [32,33,34,35], and other applications [36,37,38,39].

The recent developments in LDH nanomaterials have been significant for advancing sensor technology for environmental pollutants, biological toxins, and other substances. LDH-based sensors have shown impressive analytical performances for electrochemical and optical detection methods because of their outstanding catalytic and hosting abilities. LDHs containing more than one transition metal cation synergistically work to improve the electrocatalytic activity of LDHs; however, this behavior is not clearly understood. LDHs made up of Co (II)/Co (III) and Mn (III) cations underwent self-adjustment of Co (II) and Mn (II) oxidation states during alkaline hydrolysis while forming the octahedral state [40]. X-ray photoelectron spectroscopy (XPS) and X-ray absorption spectroscopy showed the formation of a unique phase that was stabilized by the intercalation of hydroxyl and carbonate anion along with water molecules.

The thickness of the LDH layer is very critical for sensing by electrocatalytic processes. Ni_2_Co-LDHs with a thickness of 4–6 nm (5–8 layers) prepared by a simple exfoliation method in a low boiling point solvent with carbonate ion (CO_3_^2−^) intercalants showed excellent sensing properties towards dopamine (DA) due to its low oxidation potential [41]. Density functional theory (DFT) analysis for a monolayer of the LDH was well matched with the results obtained from an experimental study and found that higher charge density was on the DA molecule, making DA a good proton acceptor. In addition to the thickness of the active LDH layer, the surface area of the exfoliated LDH gallery, conductivity, and surface charge play critical roles in defining the sensor’s efficiency. The conductivity of transition metal hydroxides is generally poor. Reduced graphene oxide (rGO), a 2D material with excellent conductivity, was added to LDHs to improve conductivity. Zn–NiAl-LDH was exfoliated in formamide and mixed with dispersed rGO in the same solvent [42]. A superlattice stacking of LDHs and rGO formed by self-assembly when freeze-dried. Although it is challenging to detect DA, uric acid (UA), and ascorbic acid (AA) simultaneously, this stacked electrode detected them very efficiently. Another way to enhance the performance of the LDH sensing of DA without following the tedious path of exfoliation is to incorporate another conducting polymeric material such as polyaniline (PANI) [43]. Charge was introduced to the electrode by amine functional groups by making a ternary system of MgAl-LDH/NiMnO/functionalized PANI, which created better interactions with organic analytes such as L-dopamine [44]. The presence of PANI enhanced the conductivity and capacitive properties, resulting in a positive influence on the redox behavior of the electrode. At the same time, the surface area of the electrode’s LDH layer increased, likely due to the intercalation of the long-chain PANI. This resulted in improved contact between the analyte and active materials. Binary metal oxides generated from the calcination of LDHs play a crucial role in the electrocatalytic properties of the electrode by enhancing the surface area and conductivity of the electrode on which another layer of LDHs was deposited [45]. Based on this concept, ZnAl-LDHs were grown on NiCoO to detect highly selectively pyridoxine. Ultrathin growths of NiFe-LDHs without exfoliating the gallery worked well for glucose detection [46]. In order to have a stronger link between the active material and the current collector, a nickel foam substrate was reacted with a cobalt precursor to prepare copper cobalt carbonate hydroxide (CCCH) [47]. The NiCo-LDHs grown on CCCH showed better efficiency in sensing glucose due to the higher conductivity of copper foam and the higher surface area of active material. The morphology of modified current collectors creates channels for electron flow and intimate contact between analyte and electrode [48]. Copper hydroxide nanotubes were grown on a glassy carbon electrode (GCE), and NiCo-LDHs were deposited onto it to prepare a core–shell structure that showed excellent sensing efficiency towards glucose. The growth of ZIF-67 nanocrystal occurred on the ultrathin, stacked NiCo-LDH by reacting 2-methylimidazole with cobalt ion. The LDHs exhibited suitable pathways for efficient electron mobility between the electrode and analyte [49]. The sensitivity of this electrode was very high—able to detect the α and β isomers of naphthol.

Optical sensors are one of the most simple and easy detection techniques. LDH-based optical detection mainly works on the principle of UV–Vis absorption, photoluminescence (PL), and chemiluminescence (CL). LDH nanomaterials have outstanding hosting capabilities for fluorophores and chromophores due to their layered structure and exchangeable anions present in the interlayers. LDHs possess enzyme-like catalytic behavior and work in combination with chromophores for colorimetric detection [50]. In order to enhance fluorescence properties, LDHs work in combination with silver nanoclusters functionalized with chromophores [51]. Platinum-coated LDHs are materials with excellent activity in combination with chromophores for colorimetric H_2_O_2_ sensing [52]. Rare earth material-functionalized LDHs improve the PL-based chemosensing ability of the electrode [53]. Rare earth materials-based double hydroxides are efficient materials for the fluorescent detection of the Fe(III) ion [54,55,56]. Layer-by-layer deposition of LDHs and dye molecules enhances the interaction between catalyst and the active molecules that enhance both electrochemical and optical sensing ability [57].

This review organizes the recent advances in LDH-based sensor designs to facilitate the development of sensitive, selective, and affordable sensors for critical biomarkers and pollutants. First, frequently used LDH synthesis methods, including co-precipitation, urea hydrolysis, ion exchange, and hydrothermal synthesis, were described. The effects of synthesis parameters, including reaction temperature, duration, and composition, were analyzed with regard to LDH structure and catalytic performance for electrochemical and optical sensor applications. Then, the advantages and disadvantages of different electrochemical and optical analysis methods were discussed. The common techniques for characterizing LDHs, measuring sensor performance, and determining analyte concentrations were also provided. Finally, the design and modifications of various LDHs and LDH composites were analyzed concerning important analytes, including glucose, DA, H_2_O_2_, metal ions, nitrogen-based toxins, and other organic molecules. The LDHs used for different analytes and methods of improving sensor performance were highlighted. As LDHs are cost-effective, easily tunable, and highly compatible with other materials, they have enormous potential as catalysts and hosts for various optical or electrochemical reactions. LDHs are promising 2D materials that have yet to be fully understood and optimized for sensor applications.

## 2. Synthesis Methods

**Figure 1:**
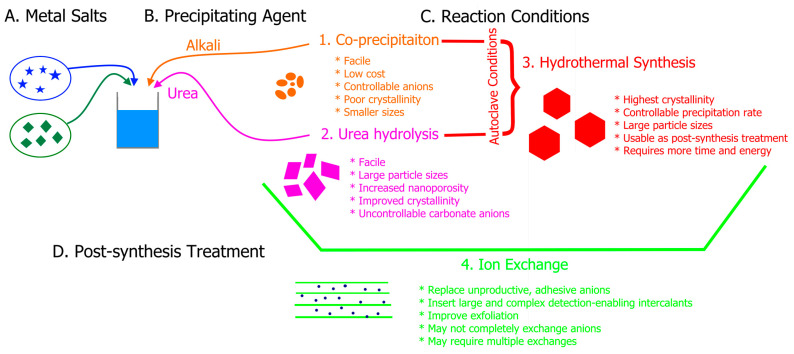
The 4 controllable synthesis parameters: (**A**) metal salts; (**B**) precipitating agent; (**C**) reaction conditions; and (**D**) post-synthesis treatments. The synthesis method corresponds to the choice of precipitating agent (co-precipitation or urea hydrolysis), reaction conditions (hydrothermal synthesis), and post-synthesis treatment (ion exchange).

LDHs have many facile synthesis methods that yield highly tunable LDH nanostructures. Four synthesis strategies include co-precipitation, urea hydrolysis, hydrothermal synthesis, and ion exchange. Figure 1 illustrates the controllable synthesis parameters (A–D) and corresponding synthesis routes (1–4), highlighting the advantages and disadvantages of each method. The general procedure, key variables, advantages, and disadvantages of each synthesis technique are discussed.

### 2.1. Co-Precipitation

Co-precipitation is one of the most frequently used LDH synthesis methods due to its low cost and simplicity. In a typical co-precipitation procedure, divalent and trivalent metal salts containing the desired cationic species are mixed in an aqueous solution at specific ratios. A soluble salt containing the specific anion species, such as Na_2_CO_3_, may be added to the metallic solution for controlled counter ion intercalation into the final LDH. The aqueous precursor is treated with an alkaline solution, such as NaOH, to raise the pH for the condensation of hexa–aqua metal complexes that produce the brucite-like LDH layers [58]. The resulting LDH exhibits a lamellar structure comprising homogeneously distributed di and trivalent metal ions with solvated counter ions. The co-precipitated LDHs may undergo thermal treatment or an aging process for improved crystallinity and higher yields [59]. The synthesized LDHs are deposited onto electrodes or other substrates for electrochemical or optical sensor applications [60].

The morphology, crystallinity, and size of LDHs can be easily controlled by choosing key experimental factors, including the M^2+^/M^3+^ metal salt ratio, atmosphere, post-treatment duration, and added anion salts. The ratio between the different metal cations in bimetallic LDHs can affect the LDH morphology and catalytic activity, owing to the unique properties of each metal. For example, in a NiCo-LDH, Co^3+^ is a hard acid that binds with 3,3′,5,5′-Tetramethylbenzidine (TMB) better than Ni^2+^, thus enabling the greater adsorption of the color-changing TMB for optical detection. Moreover, the multiple valence states of commonly used LDH metals (such as Fe and Co) can form reactive hydroxyl species following the Haber–Weiss mechanism, resulting in enhanced catalytic activity [61]. As such, Sun et al. optimized the peroxidase-like activity of NiCo–NO_3_-LDH in an H_2_O_2_-TMB system used for colorimetric H_2_O_2_ detection by increasing the Co(NO_3_)_2_ loading in the co-precipitation procedure [62]. Different Ni(NO_3_)_2_·6H_2_O and Co(NO_3_)_2_·6H_2_O were co-precipitated with NaOH at room temperature to yield ultrathin NiCo-LDH nanosheets with a ~2 nm thickness and ~26 nm lateral length. The co-precipitation reaction partially oxidized Co^2+^ to Co^3+^ and yielded LDHs with poor crystallinity. The NiCo-LDH was dispersed in water with TMB, a chromogenic compound that turns blue when oxidized by H_2_O_2_ in the presence of peroxidase or peroxidase mimics. The NiCo-LDH with a higher Co content exhibited increased nanosheet aggregation, which often reduces catalytic activity. However, the LDHs with greater Co content showed enhanced peroxidase-like ability, owing to the multivalence of Co and hard acid property of Co^3+^. Ni_0.67_Co_0.33_-LDH had the highest catalytic activity, indicated by the highest absorbance of 652 nm light when exposed to the colorimetric TMB-H_2_O_2_ system. The catalytic activity of the Ni_0.67_Co_0.33_-LDH was further optimized by adjusting the H_2_O_2_-TMB system to a pH of 5.39 and a temperature of 46 °C. The optimized NiCo-LDH sensor exhibited improved sensitivity and a lower limit of detection (LOD). Conclusively, the types of metals in multi-metal LDHs and the ratio of the involved metals can be optimized to improve the catalytic activity of LDHs for more sensitive sensors.

Interestingly, the atmosphere during co-precipitation can control the resulting counter ion adsorbed in the LDH. CO_2_ in the atmosphere, in particular, dissolves in the precipitating solution, resulting in CO_3_^2−^ intercalation [63]. In the previously discussed study by Sun et al. synthesis under regular atmosphere conditions resulted in some undesired CO_3_^2−^ intercalation into the NiCo-NO_3_-LDH. CO_3_^2−^ infiltration can occur, even if the atmosphere is carefully purged. As CO_3_^2−^ is a sticky intercalant due to its highly negative charge, CO_3_^2−^ can replace the desired counter ion responsible for the analyte detection [64]. Amini et al. found that some CO_3_^2−^ anions replaced the reactive Fe(CN)_6_^3−^ intercalants in a NiAl–Fe(CN)_6_-LDH, despite careful nitrogen purging [65]. A 2:1 molar ratio of Ni(NO_3_)_3_·6H_2_O to Al(NO_3_)_3_·9H_2_O was reacted with excess NaOH and K_3_Fe(CN)_6_ under nitrogen purging. The pH was adjusted to 9.6 with HNO_3_ and aged at 90 °C for 1 d. The NiAl–Fe(CN)_6_-LDH contained some undesirable CO_3_^2−^ intercalants despite nitrogen purging, owing to contamination with atmospheric CO_2_ during some synthesis steps. Large intercalants significantly increase the basal spacing of LDHs, demonstrated by the case with the NiAl–Fe(CN)_6_-LDH, wherein the basal spacing increased from 7.9 Å (NO_3_^−^ intercalation) to 10.46 Å spacing with Fe(CN)_6_^3−^. Larger interlayer spacing, in turn, has been found to increase CO_3_^2−^ infiltration from atmospheric CO_2_ [66], further decreasing the adsorption of the desired intercalants. The expanded brucite-like layers exerted stress on the large Fe(CN)_6_^3−^, reducing its Fe^3+^ metal centers to Fe^2+^, which exhibited poorer catalytic ability. The NiAl–Fe(CN)_6_-LDH exhibited a pure hydrotalcite-like crystal phase and aggregated to form spherical agglomerations with a diameter of ~30 nm. A terephthalic acid (TA)–H_2_O_2_ system produced a fluorescent response to the peroxidase-like NiAl–Fe(CN)_6_-LDH. The TA–H_2_O_2_ system was added to the LDH dispersion in an aqueous NaOH solution, and the fluorescence intensity of the supernatant was measured. When the TA–H_2_O_2_ system was mixed with NO_3_^−^ intercalated NiAl-LDH, the supernatant exhibited no fluorescence response. However, when the TA–H_2_O_2_ system was exposed to NiAl–Fe(CN)_6_-LDH, the supernatant emitted intense light peaking at 422 nm. Thus, the Fe(CN)_6_ was solely responsible for the peroxidase-like reactivity. Cr^3+^ and Cr^4+^ oxidized the Fe^2+^, resulting in increased peroxidase-like activity, indicated by more intense fluorescent responses. Thus, increasing the amount of initial Fe(CN)_6_^3−^ intercalation by reducing CO_3_^2−^ adsorption would increase the amount of Fe^2+^ to oxidize, increasing the Cr detection range. While the co-precipitation method is simple and cost-effective, the synthesis route introduces undesirable CO_3_^2−^ intercalation from the atmosphere, reducing LDH sensor performance, especially if the intercalant is responsible for the analyte detection.

If the intercalant does not significantly affect analyte detection, CO_3_^2−^ intercalation may be desirable as it yields a compact lamellar structure and a more negative surface charge. Zhu et al. intercalated CO_3_^2−^ in NiAl-LDH for electrochemical DA detection [67]. Ni(II) and Al(III) in a ratio of 3:1 were dissolved in CO_3_^2−^-free water and treated with NaOH and Na_2_CO_3_ under constant stirring. The NiAl–CO_3_-LDH exhibited a mostly uniform, flake-like nanostructure with diameters between 20 and 40 nm and a thickness of several nanometers. The addition of Na_2_CO_3_ during synthesis resulted in charge-balancing CO_3_^2−^ intercalation. The highly negatively charged CO_3_^2−^ keeps the positively charged brucite-like layers together, increasing LDH stability, indicated in the case of NiAl–CO_3_-LDH with little change to its nanostructure after more than 6 months in aqueous suspension. For electrochemical detection, a traditional carbon paste electrode (CPE), comprising a homogeneous graphite and liquid paraffin mixture, was modified into a carbon ionic liquid electrode (CILE). The CILE was synthesized by grinding graphite powder and liquid paraffin with 1-(3-chlorine-2- hydroxypropyl)-3-methyl-imidazolium acetate. The resulting homogeneous paste was tightly packed into an open-ended glass tube. A well-dispersed solution of NiAl-LDH was deposited onto the CILE surface and dried at room temperature, yielding a thin film on the CILE. The small size of the intercalated CO_3_^2−^ enables the synthesis of more compact LDH nanosheets for increased surface area. The tiny NiAl–CO_3_-LDH increased the surface area of the CILE, allowing greater adsorption of DA. The LDH-modified CILE exhibited a 2.1-times-higher peak redox current for DA than bare CILE, owing to the high porosity of the LDH film that enabled fast DA adsorption. In a neutral phosphate-buffered solution (PBS), the LDH exhibited a negative surface charge, likely due to the highly negatively charged CO_3_^2−^ counter ions. The negatively charged NiAl-LDH attracted the positively charged DA and repelled the negatively charged ascorbic acid for improved sensor sensitivity and reduced interference. CO_3_^2−^ intercalation during co-precipitation yields compact LDHs that increase the surface area of electrodes and endow a negative surface charge for improved analyte adsorption, resulting in enhanced electrochemical detection.

Lastly, co-precipitated LDHs can undergo an aging or thermal process for improved crystallinity. LDHs synthesized via co-precipitation often exhibit poor crystallinity due to the rapid phase change in solution, resulting in reduced electron mobility during catalysis and electrochemical redox process. A facile method of improving crystallinity is to age the precipitated LDH dispersion. Asadpour-Zenynali et al. improved the crystallinity of NiAl–Fe(CN)_6_-LDH by aging the LDHs for 24 h, yielding excellent detectors for the electrochemical detection of paracetamol [68]. An aqueous solution with a 2:1 molar ratio of Ni(NO_3_)_3_·6H_2_O to Al(NO_3_)_3_·9H_2_O was treated with NaOH and excess K_3_Fe(CN)_6_ under vigorous stirring in a nitrogen atmosphere to a pH of 9.6. Increasing the aging duration improved the crystallinity of the LDH nanospheres. The Fe(CN)_6_-intercalated LDHs comprised nanoparticles smaller than 100 nm diameter and an expanded basal spacing of 1.046 nm. Similar to the previously analyzed work by Amini et al., some Fe^3+^ was reduced to Fe^2+^, due to stress from the lamellar structure [65]. The NiAl–Fe(CN)_6_-LDH was drop-cast onto a polished GCE, yielding a thin LDH film. Unlike the NO_3_^−^ or CO_3_^−−^ intercalants, the Fe^3+^ centers in Fe(CN)_6_ could oxidize paracetamol for an electrochemical signal. The highly crystalline LDHs effectively oxidized Fe^2+^ to Fe^3+^ for increased paracetamol oxidation. Thus, the NiAl–Fe(CN)_6_-LDH exhibited an almost 2-fold higher peak oxidation current than the Fe(CN)_6_-less LDH sensor. LDH crystallinity can also improve after hydrothermal treatment, as discussed more thoroughly in Section 2.3. Briefly, the high temperature and additional aging time allow LDH nanoparticles to collide under high pressures that facilitate crystal growth. Silva et al. found that LDHs synthesized via co-precipitation exhibited poor crystallinity compared to LDHs synthesized via microwave or hydrothermal methods, owing to the lack of high temperature, pressure, and aging time [69]. The crystallinity of co-precipitated MgFe-LDHs was increased by aging the LDHs under constant agitation for 4 h at room temperature followed by hydrothermal treatment in an autoclave at 160 °C for 16 h. Both aging and thermal treatment take additional time and energy, which may not be necessary for LDHs in sensors used for specific analytes at higher detection ranges.

The co-precipitation method is the most commonly used LDH synthesis process, owing to its facile procedure, low cost, and ambient reaction conditions. The morphology, intercalated anions, crystallinity, and particle size of the resulting LDHs to a certain degree by adjusting the metal salt ratio, adding dopants, and post-synthesis thermal treatment/aging. However, the co-precipitation method often yields small, aggregated LDHs with poor crystallinity due to its rapid reaction with strong alkali precipitating agents. Moreover, atmospheric CO_2_ may hinder the intercalation of the desired counter ion required for the sensing mechanism.

### 2.2. Urea Hydrolysis

The urea hydrolysis is a facile LDH synthesis method similar to co-precipitation that uses urea as the primary precipitating agent instead of NaOH or other common precipitating agents [70]. Unlike the co-precipitation method, urea hydrolysis yields LDHs with primarily CO_3_^2−^ counter ions, regardless of the initial metal salts and atmospheric conditions. Generally, different molar ratios of M^2+^/M^3+^ metal salts are treated in solution with urea, wherein the reaction temperature and solubility are adjusted (often under hydrothermal conditions) to control the urea hydrolysis rate, yielding LDHs with controllable size, morphology, and crystallinity [71]. The ability to control the urea hydrolysis rate is especially useful for synthesizing LDHs with greater crystallinity and uniformity than through the co-precipitation method [72].

The greatest disadvantage of urea hydrolysis is the inability to choose the primary counter ion adsorbed by the LDHs, owing to the dominance of charge-balancing CO_3_^2−^ as a byproduct of urea decomposition [73]. If CO_3_^2−^ is the desired intercalated anion, then urea hydrolysis becomes a more facile method than co-precipitation as no careful atmospheric control nor additional NaCO_3_ is required. Ni et al. utilized urea hydrolysis to synthesize thin NiCo–CO_3_-LDH nanosheets composited with polypyrrole for electrochemical glucose detection [74]. A solution Co(NO_3_)_2_ to Ni(NO_3_)_2_ was treated with excess urea in a water/ethylene glycol solution. The synthesized CoNi-LDH exhibited a nanosheet morphology with irregular shapes, deviating from the typical hexagonal structure. The LDH possessed a hydrotalcite-like crystal phase with interlayer CO_3_^2−^. The CoNi-LDH was stirred in a FeCl_3_ solution and mixed with a pyrrole solution in a blue-cap bottle at 50 °C for 2 days to polymerize the polypyrrole shell. The CoNi-LDHs retained their nanosheet morphology, owing to the polypyrrole forming a thin-film coating on the edges of the LDH. The CoNi-LDH/polypyrrole was dispersed in a Nafion solution and drop-casted onto a polished GCE. Glucose oxidation into gluconolactone was electrochemically detected as the glucose reduced the Ni and Co metal centers. Thus, the CO_3_^2−^ intercalants did not directly participate in aiding or hindering the electrochemical detection process. Rather, the slow 3 h urea precipitation process yielded thin and large 1 μm nanosheets for thin and uniform polypyrrole coating. The conductive polypyrrole shell enhanced electron transfer to the CoNi-LDHs, resulting in a low 62.32 Ω charge transfer resistance with polypyrrole compared to 121 Ω without polypyrrole. The polypyrrole-modified LDH exhibited a 10-fold higher and 6-fold higher peak oxidation current with and without glucose exposure, respectively. Thus, if the intercalated anion is not involved in the primary detection mechanism, urea hydrolysis is a more simple and controllable alternative to co-precipitation.

Using urea instead of strong bases, such as NaOH, allows for more ideal crystallization conditions for stable LDH nanosheet growth. The decomposition of urea leads to CO_3_^2−^ products that intercalate in the LDH sheets, leaving NH_4_^+^ to raise the pH to around 9 for uniform LDH precipitation [75]. Moreover, the NH_4_^+^ acts as nucleation sites for LDH crystallization to further accelerate LDH formation. The stability of urea hydrolysis was emphasized in a study by Li et al., wherein NiAl-LDHs were directly synthesized onto Ti foil using urea, whereas NaOH failed [76]. Ti foil was etched with hydrofluoric acid and washed with distilled water. Urea was added to a solution containing Ni(NO_3_)_2_ and Al(NO_3_)_3_, and the treated Ti foil was vertically submerged in the solution, yielding Ti foil coated with a NiAl-LDH thin-film. The decomposed urea solution reached a steady pH of about 9 for optimal precipitation, and its slow decomposition provided NH_4_^+^ that facilitated LDH nucleation, resulting in a uniform film coating. The NiAl-LDH film comprised interconnected, vertically aligned nanosheets with a 10–20 nm thickness and 300–600 nm lateral length. A similar co-precipitation procedure with NaOH precipitant did not yield the uniform film coating, likely owing to the rapid and unfacilitated precipitation process. The porous NiAl-LDHs afforded effective analyte adsorption for increased electrode sensitivity and catalytic activity. Bare Ti-foil exhibited no oxidation current when exposed to glucose. However, the NiAl-LDH-modified Ti foil exhibited a distinct peak oxidation current, owing to the catalytic Ni centers that oxidized glucose. The same urea hydrolysis process was repeated with glass and graphite substrates. The glass substrate was too smooth for strong LDH nucleation, resulting in poor film adhesion. The graphite substrate had improved film adhesion, but the growth of NiAl-LDH nanosheets was non-uniform, owing to the graphite’s uneven surface. The binder-less electrochemical probe fabrication method yields electrodes with higher conductivity for more sensitive and lower limit electrochemical sensing. The urea hydrolysis method can afford the stability to directly synthesize LDHs onto conductive surfaces with controlled precipitation conditions and NH_4_^+^-facilitated nucleation.

The stable and controlled crystallization via urea hydrolysis is emphasized by the larger LDHs compared to other synthesis methods. The mild reaction conditions and long reaction durations facilitate the growth of large, crystalline LDH nanosheets. These larger nanosheet structures enhance the sensing ability for detection mechanisms that rely on the intercalated species. Wang et al. found that MgAl-LDHs synthesized via urea hydrolysis were significantly larger and more responsive for amine detection than MgAl-LDHs synthesized via co-precipitation [77]. A solution of Mg(NO_3_)_2_·6H_2_O to Al(NO_3_)_3_·9H_2_O was treated with urea at 100 °C in a Teflon-lined autoclave. The MgAl–CO_3_-LDH exhibited a regular hexagonal shape and lamellar nanosheet morphology with a large 2 μm diameter. The intercalated charge-balancing CO_3_ was replaced with NO_3_^−^ via salt–acid ion exchange (see Section 2.4) and subsequently dispersed in formamide. The hydrogen bonding between the hydroxyl groups in the MgAl-LDHs and formamide likely led to the strong blue emissions under UV (365 nm) irradiation. The urea hydrolysis-synthesized LDH exhibited the most intense CL emissions, owing to the larger nanosheet size affording more stable hydrogen bonding between the formamide and LDH. The Mg-to-Al ratio was also easily controlled by changing the initial metal salt loadings. Increasing Mg content decreased the positive charge of the brucite-like layers, decreasing the interactions with leftover CO_3_^2−^ anions, enabling more hydrogen bonding with the formamide. Exposure to amines displaced the hydrogen bonding between formamide and the MgAl-LDH, resulting in decreased fluorescence intensity. The exfoliated MgAl–NO_3_-LDH was added to a bis(2,4,6-trichlorophenyl) oxalate (TCPO)-H_2_O_2_ CL system for more sensitive amine detection. Formamide reacted with TCPO to form *OOH radicals that facilitated the conversion of TCPO into 2,4,6-trichlorophenol. The combination of MgAl-LDH and formamide exhibited enhanced catalytic activity, indicated by the ~10-times-greater CL intensity from the TCPO–H_2_O_2_ CL system, enabling a lower LOD. Urea hydrolysis yields exceptionally large, uniform LDH nanosheets that can host many detection-facilitating intercalants, resulting in improved sensor sensitivity and lower LOD.

By controlling the reaction temperature of urea hydrolysis, complex LDH morphologies can be synthesized. Higher temperatures expedite urea decomposition for more rapid LDH nucleation, yielding smaller nanosheets structures. The abundant NH_4_^+^ from decomposed urea become nucleation centers for more unusual nanosheet arrangements, unlike the common nanosphere aggregations from co-precipitation [68]. More complex nanostructures, such as nanoflowers, offer greater surface area and structural stability than lamellar nanosheet aggregations for greater analyte adsorption and sensor stability. Chen et al. used controlled urea hydrolysis to synthesize porous NiAl-LDH nanoflowers for electrochemical glucose detection [78]. A solution containing a 2:1 ratio of Ni(NO_3_)_3_·6H_2_O to Al(NO_3_)_3_·9H_2_O was treated with urea at 100 °C. The high temperature and agitation would yield smaller nanoparticle sizes but more nucleation points. The solution was subsequently aged at 94 °C for 14 h, allowing the nucleated LDHs to grow into larger nanosheets that formed the nanoflower petals. The NiAl-LDH precipitate was collected via centrifugation, rinsed with deionized water and ethanol, and air-dried, yielding a NiAl-LDH powder. The NiAl-LDH possessed a nanoflower-like morphology comprised of interconnected LDH nanosheets, with a 10–20 nm thickness and 600 nm lateral length. The NiAl-LDH was dispersed in a Nafion/ethanol solution and drop-casted onto a polished GCE. The NiAl-LDH/Nafion/GCE was air-dried, yielding a catalytic NiAl-LDH/Nafion thin-film layer. The Ni centers in the NiAl-LDH oxidized glucose into gluconolactone, which was detected as a large peak reduction current. The high surface area promoted abundant glucose adsorption for a wider detection range and lower LOD. The interconnected nanoflower structure facilitated electron transfer, leading to stronger electrochemical signals for enhanced glucose sensitivity. The urea hydrolysis method should be used for synthesizing complex nanostructures as the hydrolysis rate and aging duration can be easily controlled with temperature and time.

LDH synthesis via the urea hydrolysis method is a popular alternative to the co-precipitation method because the LDH particle size, crystallinity, and morphology can be easily controlled by adjusting the urea decomposition rate. The biggest disadvantage of urea hydrolysis is the guaranteed, adhesive CO_3_^2−^ intercalants, which may be an issue if the CO_3_^2−^ needs to be replaced with specific detection-facilitating anions. However, if the intercalated anions are not required for the detection mechanism, the urea hydrolysis method is an excellent choice for large LDHs with high porosity for sensor applications.

### 2.3. Hydro(solvo)thermal Synthesis

The hydrothermal synthesis process involves a reaction in an aqueous solution under autoclave conditions at high temperatures above the boiling point of water. Hydrothermal synthesis procedures reported in the literature often involve performing the co-precipitation or urea hydrolysis methods under hydrothermal conditions [79]. The solvothermal method is analogous to the hydrothermal method but involves reactions in an organic solvent [80]. For LDHs, there are two primary applications for hydrothermal/solvothermal synthesis: directly synthesizing LDHs using precursor materials or the post-synthesis treatment of already-synthesized LDHs. The reaction temperature, duration, and initial metal salt ratios can be easily controlled to yield the desired morphology, size, and crystallinity [81].

The direct hydrothermal LDH synthesis process is initially similar to co-precipitation and urea hydrolysis, wherein aqueous metal salts are mixed in specific ratios and treated with precipitating agents such as alkali or urea. However, before the typical co-precipitation or urea hydrolysis is allowed to be complete, the mixture is transferred to a stainless-steel Teflon-lined autoclave for high pressure and temperature reaction conditions. Liu et al. synthesized a MgAl-LDH via direct hydrothermal co-precipitation reaction and subsequently doped the MgAl-LDHs with L-glutathione–Mn–ZnS quantum dots (GMZS QDs) for heavy metal ion detection [82]. The stable crystal growth under hydrothermal conditions yielded large 400 nm nanosheets that facilitated GMZS QDs adsorption. An aqueous solution containing MgCl_2_·6H_2_O and AlCl_3_·6H_2_O was treated with Na_2_CO_3_. The pH of the solution was adjusted to 12 with NaOH for hydrothermal treatment. The MgAl-LDH comprised a disk-like morphology with a 30 nm thickness, 400 nm diameter, and excellent crystallinity. GMZS QDs were dispersed in deionized water and treated with the MgAl-LDH under constant stirring for 5 h at room temperature. Three-nanometer diameter QDs were uniformly adsorbed onto the LDH, owing to the electrostatic interaction between the positively charged brucite-like layer and the negatively charged L-glutathione-Mn–ZnS QDs. The high specific surface area afforded by the lamellar nanosheet structure allowed for abundant QD adsorption. The GMZS QDs exhibited luminescence under 320 nm irradiation. Increasing the QD content resulted in decreased luminescence intensity, owing to unproductive QD agglomerations at high QD concentrations. The LDH provided thermal and chemical stability by immobilizing the QDs for reduced aggregation and enhanced luminescent response, allowing higher QDs loading without aggregation. Exposure to Pb^2+^, Cr^3+^, and Hg^2+^ resulted in decreased luminescence intensity due to various QD quenching mechanisms. The hydrothermal method yields large, uniformly sized, and highly crystalline LDHs that provide a stable structure for detection-enabling host molecules.

The most critical variables to control during hydrothermal synthesis include the metal salt ratios, reaction temperature, and treatment duration. Just as with the co-precipitation and urea hydrolysis methods, the metal content of the different metals in LDHs can be controlled by adjusting the initial ratio of metal salts mixed. However, the ratio of the added metal salts may not result in the same metal ratios in the resulting LDHs, thus requiring experimental optimization [83]. The metal ratios in the final LDHs heavily influence their morphology, and consequently, their performance as sensors. Wang et al. controlled the Co-to-Al ratio in CoAl-LDHs synthesized via hydrolysis for optimal electrochemical NO_x_ detection [84]. Aqueous solutions with varying ratios of Co(NO_3_)_2_·6H_2_O and Al(NO_3_)_3_·9H_2_O were treated with urea for hydrothermal treatment. The resulting CoAl–CO_3_-LDH exhibited high crystallinity with an interlayer spacing of 0.767 nm. All Co:Al ratio variations of the LDH comprised a thin nanosheet morphology, but a 2:1 Co:Al ratio (Co_2_Al-LDH) resulted in the most homogeneous distribution of regular hexagon-shaped nanosheets with a 19.3 nm lateral length. A low Co:Al ratio of 1:1 resulted in irregularly shaped nanosheets, whereas a Co:Al ratio of 3 resulted in significant nanosheet aggregation. The Co_2_Al-LDH exhibited the highest BET surface area of 54 m^2^ g^−1^, which afforded maximum NO_x_ gas adsorption. The Co_2_Al-LDH also possessed 2–5 nm-diameter mesopores in the nanosheet surface, owing to the ammonia and CO_2_ formed from urea decomposition. The thermal control afforded by the hydrothermal process enabled the urea to slowly hydrolyze into ammonium hydroxide and CO_2_, which was essential for precipitation and pore formation. Higher temperatures resulted in faster urea decomposition for more pore-forming agents, resulting in higher LDH surface area for gas absorption. A Co_2_Al-LDH suspension was drop-cast onto a clean Au electrode, yielding an LDH thin-film probe. The high adsorptive ability of Co_2_Al-LDH resulted in a sensitive response to NO_x_ exposure and the short response and recovery times. The hydrothermal process is a facile method wherein the temperature and metal ratios can be easily controlled for optimal LDH morphologies.

In another example, Liu et al. varied the initial ratio of the divalent and trivalent metal nitrate precursors to synthesize an optimal CoAl-LDH for electrochemical NO_2_ gas detection [85]. Aqueous solutions containing varying ratios of Co(NO_3_)_2_·6H_2_O and Al(NO_3_)_3_·9H_2_O were treated with urea and NH_4_F under hydrothermal conditions. The CoAl-LDH nanosheets aggregated to form a microflower morphology. The F^−^ functioned as a templating agent that directed the formation of the interconnected 2–4 nm-long nanosheet structure. The CoAl-LDH exhibited a high degree of crystallinity with intercalated CO_3_^2−^. Changing the ratio of divalent and trivalent metal salt precursors in the hydrothermal process did not yield the same divalent to trivalent metal ratio in the resulting LDH. CoAl-LDH synthesized with a 1:1 mole ratio of Co(NO_3_)_2_·6H_2_O to Al(NO_3_)_3_·9H_2_O produced a 3.4:1 molar ratio of Co to Al, resulting in incompletely formed LDH nanosheets. However, the CoAl-LDH synthesized with a 3:1 metal nitrate ratio possessed a 3.8:1 Co-to-Al ratio, which resulted in excess urchin-like morphology that occupied the interstitial space of the main nanoflower structure. The CoAl-LDH synthesized with a 2:1 initial metal nitrate ratio (Co_2_Al-LDH) exhibited a 3.65:1 ratio of Co to Al, yielding 2.4–3.4 μm-wide microflower morphology comprising fully formed 3 nm-thick LDH nanosheets without the urchin-like obstructions. Thus, the Co_2_Al-LDH exhibited the largest BET surface area of 49.45 m^2^ g^−1^. The large specific surface area of the Co_2_Al-LDH resulted in excellent NO_2_ adsorption/desorption capability, indicated by its fast response and recovery times when exposed to 100 ppm NO_2_ gas and fresh air, respectively. The hydrothermal process enables the controlled synthesis of LDH with varying divalent and trivalent metal compositions for an optimal LDH morphology that maximizes analyte adsorption.

The hydrothermal reaction temperature and duration have similar effects on the resulting morphology and crystallinity of LDHs. Zhang et al. found that low temperatures below 100 °C did not yield any significant MgAl-LDH crystal growth, whereas increasing the temperature to 160 °C resulted in highly crystalline nanospheres with well-defined flower-like nanosheet growths [86]. The 100 °C LDHs exhibited smaller spherical sizes of 2.5 μm than the 4 μm diameters of LDHs synthesized at 140 °C. Increasing the reaction time yielded similar results, with short 1 h reactions producing few LDH nanopetals and longer 6 h reactions yielding well-defined nanoflower morphologies. The hydrothermal synthesis parameters also controlled the dispersion of the MgAl-LDHs, with higher temperatures and longer reaction times resulting in uniform distribution due to the formation of highly crystalline nanostructures. The key hydrothermal parameters can be easily tuned to yield the desired morphology for LDH sensor applications.

The hydrothermal method can also improve the crystallinity and sizes of LDHs synthesized via co-precipitation or urea hydrolysis. Li et al. heat-treated NiFe-LDHs synthesized via urea hydrolysis under hydrothermal conditions for 2 days, resulting in large nanosheet growths around 500 nm. They synthesized an 8-hydroxypyrene-1,3,6-trisulfonicacid trisodium (HPTS)/NiFe-LDH hybrid material for fluorescent CO_2_ detection via a one-pot hydrothermal process [87]. Ni(NO_3_)_2_·6H_2_O, Fe(NO_3_)_3_·9H_2_O, urea, and trisodium citrate were added to an HPTS solution under a nitrogen atmosphere. The mixture underwent hydrothermal heat-treatment at 150 °C for 2 days, yielding an HPTS/NiFe–CO_3_-LDH composite precipitate. The hydrothermal conditions provided a stable environment for good crystallization, yielding large, uniform hexagonal platelets. The composite was collected via filtration, washed with decarbonated water, and air-dried. The HPTS/NiFe-LDH exhibited an increased basal spacing of 0.91 nm, indicating the intercalation of the fluorescent HPTS dye. HPTS-less LDH comprised typical hexagonal nanoflakes. However, HPTS-LDH exhibited an aggregated nanoflower morphology formed from interconnected LDH nanosheets. Increasing the initial HPTS content from 11 to 491 mg resulted in increased LDH nanoflower agglomerations. The HPTS/NiAl-LDH with 98 mg of initial HPTS was dispersed in ultrapure water with ultrasonication for 2 h. The composite exhibited no fluorescence under 402 nm irradiation because there was insignificant free-floating HPTS in the solution. Bubbling CO_2_ into the solution created CO_3_^2−^ that displaced the HPTS from the LDH gallery into the solution, increasing the fluorescence intensity. Increased CO_2_ expelled more HPTS into the solution, enabling increased light absorption and higher fluorescence intensity. The hydrothermal process method is a facile post-synthesis treatment method for increasing crystallinity and LDH particle sizes.

Some of the largest drawbacks of the hydrothermal method include the high temperature, long duration, and production of wastewater. The disadvantages of the hydrothermal process can be reduced by applying the milling procedure used in the mechanochemical LDH synthesis method [88]. In a typical mechanochemical procedure, different anhydrous metal hydroxides or nitrates, such as Mg(OH)_2_ and Al(NO_3_)_3_, are milled into a homogeneous mixture in a planetary ball mill. Subsequently, the mixture is milled again in the presence of water to yield the LDHs [89]. CO_3_^2−^ intercalation from the atmosphere can be reduced by replacing water with aqueous metal nitrates [90] or sodium hydroxide pellets [91] in the second milling step. While the mechanochemical method is facile and environmentally friendly without the use of solvents, the synthesized LDHs often exhibit poor crystallinity and significant aggregation [92]. Hydrothermal treatment has been used to overcome the drawbacks of the mechanochemical method in a process called mechano-hydrothermal synthesis. Zhang et al. synthesized trimetallic MgAlFe–NO_3_-LDHs via the mechano-hydrothermal method. Anhydrous Mg(OH)_2_ and Al(OH)_3_ were milled for 1 h in a planetary ball mill and subsequently heated in a Teflon-lined autoclave with aqueous Fe(NO_3_)_3_ [93]. The resulting hexagonal MgAlFe–NO_3_-LDH nanosheets exhibited improved crystallinity and dispersion than LDHs synthesized following a typical mechanochemical process. The surface area of the mechano-hydrothermal LDHs was more than 12-times-higher at 83.2 m^2^ g^−1^ than the mechanochemical LDHs. A purely hydrothermal reaction between Mg(OH)_2_, Al(OH)_3_, and Fe(NO_3_) did not yield LDHs at 80 °C after 12 h, suggesting that the initial milling effectively lowered the required temperature for LDH formation. In a follow-up study, Zhang et al. intercalated dodecyl sulfate (DS) into a MgAl-LDH via the mechano-hydrothermal method [94]. Anhydrous Mg(OH)_2_ and Al(OH)_3_ were milled and reacted with DS in a Teflon-lined autoclave at different temperatures and durations. Temperatures below 120 °C and reaction durations under 24 h yielded incomplete MgAl–DS-LDHs. Mechano-hydrothermal synthesis at 120 °C for 24 h yielded highly crystalline nanosheet aggregations due to the DS intercalation, whereas pure MgAl–NO_3_-LDHs comprised well-dispersed hexagonal nanosheets. The pure hydrothermal reaction without prior metal hydroxide milling under the same conditions did not yield LDHs.

The hydrothermal synthesis method often involves performing the common co-precipitation or urea hydrolysis reactions under high pressure and temperature in an autoclave. The hydrothermal method allows for finer control over the precipitation rate via changing the hydrothermal reaction temperature and duration, resulting in LDHs with high crystallinity and large particle sizes. The hydrothermal conditions may also be used as a post-synthesis aging and heat treatment method to improve the crystallinity of already-synthesized LDHs. The greatest drawbacks of the hydrothermal process are the large energy consumption for maintaining a high temperature and long reaction duration. These disadvantages may be minimized by introducing a mechanical milling step used in mechanochemical synthesis before the hydrothermal reaction.

### 2.4. Ion Exchange

The ion exchange process is a frequently used post-synthesis treatment method that replaces adhesive charge-balancing ions, such as NO_3_^−^, CO_3_^2−^, and Cl^−^, with specific anions by adding an excess of the desired anion species to the LDHs in solution and vigorously agitating the solution under a nitrogen atmosphere [95]. As most simple LDH synthesis methods, such as co-precipitation and urea hydrolysis, yield tightly bound NO_3_^−^ or CO_3_^2−^ anions that may not contribute to the sensor detection mechanism, the ion exchange method replaces unproductive intercalants with specialized detection-enabling intercalants [96].

The ion exchange process is often required to replace CO_3_^2−^ and NO_3_^−^, which have a high affinity for the brucite-like layers, with large and complex counter ions that can improve the LDHs’ properties for improved sensor performance. Asadpour-Zeynali et al. replaced intercalated NO_3_^−^ with thioglycolic acid (TA) in a MgAl-LDH for electrochemical Hg^2+^ detection [97]. MgAl–NO_3_-LDH was first synthesized via the co-precipitation of Mg(NO_3_)_2_·6H_2_O and Al(NO_3_)_3_·9H_2_O under inert alkaline conditions. The MgAl–NO_3_-LDHs were treated dropwise with excess TA to a pH to 8 and refluxed at 60 °C for 18 h under a nitrogen atmosphere. The resulting MgAl–TA-LDHs exhibited a hydrotalcite-like crystal phase and increased basal spacing from 7.6 to 10.1 Å for NO_3_^−^ intercalated to TA-intercalated LDH, respectively. The MgAl–TA-LDHs comprised 100 nm diameter nanospheres that aggregated to form a plate-like morphology. The LDHs were drop-cast onto a GCE, yielding a MgAl–TA-LDH thin film. The TA-intercalated LDH exhibited a higher anodic peak current than the NO_3_-intercalated LDH. The thiol groups in the TA chelated with the Hg^2+^, resulting in an increased surface adsorption of the Hg^2+^ onto the LDH. The peak response current of the MgAl–TA-LDH was further optimized by adjusting the pH to 4, the potential to −0.7 V, and the Hg^2+^ exposure time to 400 s. Higher Hg^2+^ content increased the peak response current, owing to increased Hg^2+^ oxidation. Ion exchange enables the synthesis of LDHs with larger charge-balancing anions that improve analyte adsorption for more sensitive electrochemical detection.

The ion exchange process is frequently used to replace optically unreactive anions with light-interacting molecules for optical analyte detection. Abdolmohammed-Zadeh et al. synthesized MgAl-LDH nanosheets with intercalated fluorescent salicylic acid (SA) for fluorometric Fe^3+^ detection [98]. The MgAl-LDH was first synthesized via co-precipitation, wherein MgCl_2_ and AlCl_3_ were treated with NaOH and NaCl under nitrogen purging. The pH was adjusted to 10 with additional NaOH, yielding a MgAl–Cl-LDH. The MgAl–Cl-LDH was dispersed in an aqueous SA solution and agitated for a day under an inert atmosphere, yielding a MgAl–SA-LDH which exhibited an increased interlayer spacing of 10.92 Å. The MgAl–SA-LDH nanomaterial comprised agglomerations of thin, 50–150 nm hexagonal nanoflakes. Ion exchange with higher concentrations of SA up to 100 mg L^−1^ enhanced the fluorescence intensity of the MgAl–SA-LDH when exposed to Fe^3+^. However, SA concentrations greater than 100 mg L^−1^ did not increase the fluorescence intensity, suggesting SA saturation. The fluorescent response of the SA-LDH was further optimized by adjusting the pH to 7 and the temperature to 25 °C. Increasing Fe^3+^ content decreased the fluorescent intensity due to the formation of nonradiative SA–Fe complexes. The MgAl–SA-LDH exhibited significantly higher fluorescence intensities than aqueous SA, owing to reduced collision quenching afforded by the homogeneous immobilization of SA in the LDH matrix. Ion exchange enables the complete intercalation of light-interacting anions for optical analyte detection.

Ion exchange is one of the only methods of replacing the highly complexing CO_3_^2−^ anion byproducts of LDHs synthesized via urea hydrolysis. As CO_3_^2−^ anions are small and highly negatively charged, they tightly hold onto the LDH layers, making LDHs difficult to exfoliate and manipulate. Xu et al. replaced a ZnAl-LDH’s interlayer CO_3_^2−^ with NO_3_^−^ for more effective LDH exfoliation in formamide [99]. ZnAl–CO_3_-LDH was first synthesized via urea hydrolysis of Zn(NO_3_)_2_·6H_2_O and Al(NO_3_)_3_·9H_2_O. The resulting ZnAl–CO_3_-LDHs were dispersed in an HNO_3_/methanol solution and agitated for 3–5 h under nitrogen purging at room temperature, yielding a ZnAl–NO_3_-LDH. The gallery height of the LDH increased from 0.75 nm with CO_3_^2^^−^ to 0.89 nm with NO_3_^−^. With decreased interlayer attraction and large basal spacing afforded by the NO_3_^−^ intercalant, the ZnAl–NO_3_-LDH was effectively exfoliated in formamide. The NO_3_-intercalated LDH was dispersed in a formamide solution, shaken for 3 days, and centrifuged, yielding a colloidal suspension of ZnAl-LDH. An acid-treated quartz substrate was dip-coated with the ZnAl-LDHs and a polyaniline (PANI) solution. The alternating dip-coating processes produced a multilayer ZnAl-LDH/PANI probe with 2 nm-thick bilayers for electrochemical ammonia gas detection. Adding bilayers increased the film thickness and roughness. The increase in surface roughness resulted in greater sensitivity to ammonia gas.

Multiple ion exchange steps may be performed to replace the original interlayer anion. Zhan et al. employed two ion exchange steps to convert Ni_2_Al–CO_3_-LDHs into Ni_2_Al–NO_3_-LDHs for electrochemical bisphenol A detection [100]. Ni_2_Al–CO_3_-LDHs were first synthesized via urea hydrolysis. The salt–acid ion exchange method was utilized to replace CO_3_^2^^−^ with Cl^−^, wherein the Ni_2_Al–CO_3_-LDHs were dispersed in a solution of excess NaCl and HCl and agitated, resulting in a Ni_2_Al–Cl-LDH. The Cl-intercalated LDHs were then dispersed in a NaNO_3_ solution and agitated, yielding a Ni_2_Al–NO_3_-LDH. The basal spacing increased from 0.758 to 0.882 nm after ion exchange from CO_3_^2^^−^ to NO_3_^−^ intercalant. The Ni_2_Al–NO_3_-LDHs retained the regular hexagonal nanoflake morphology of the Ni_2_Al–CO_3-_LDHs with a 1–2 μm lateral size and pure hydrotalcite-like crystal phase. The Ni_2_Al–NO_3_-LDHs were exfoliated in an aqueous L-aspargine solution under a nitrogen atmosphere. The exfoliated Ni_2_Al-LDH comprised irregularly shaped ultrathin nanosheets with 200 nm lateral lengths. The exfoliated LDH exhibited a significantly higher peak oxidation current than bulk Ni_2_AL-LDH due to the large surface area exposing abundant active sites. The enhanced accessibility to the positive LDH brucite-like layer also facilitated bisphenol A adsorption for improved catalytic activity.

Similarly, Li et al. used two ion exchange steps to replace interlayer CO_3_^2^^−^ with NO_3_^−^ in a MgAl-LDH [101]. MgAl–CO_3_-LDH was first synthesized via co-precipitation, wherein a solution of Mg(NO_3_)_2_·6H_2_O and Al(NO_3_)_3_·9H_2_O was treated with ammonia, yielding MgAl–CO_3_-LDHs. The salt–acid method was used to replace CO_3_^2−^ with Cl^−^, wherein the CO_3_-LDH was mixed with NaCl and HCl, sealed in a reactor filled with a nitrogen atmosphere, and shaken for two days. The resulting MgAl–Cl-LDH was then dispersed in an HNO_3_ solution for ion exchange into MgAl–NO_3_-LDH. Replacing the CO_3_^2−^ was essential for exfoliation as intercalated CO_3_^2−^ exhibits the strongest intermolecular attraction for the LDH layers [101]. The MgAl–NO_3_-LDH was effectively exfoliated in formamide owing to the weaker interlayer attraction afforded by the intercalated NO_3_. Ion exchange is a versatile technique that can be used multiple times to replace charge-balancing anions for more effective LDH exfoliation.

While the ion exchange method is not in itself an LDH synthesis method, it is one of the most common methods of replacing the intercalated anions of LDHs with specific anions involved in optical or electrochemical detection. Ion exchange is especially useful in replacing common CO_3_^2−^ and NO_3_^−^ intercalants that exhibit a high affinity for the brucite-like layers. Exchanging adhesive anions with larger and less attractive counter ions improves LDH exfoliation for an increased surface area. Multiple ion exchange steps may be used to intercalate large or complex charge balancing anions.

## 3. LDH Characterization and Analyte Detection

LDHs are promising materials for both electrochemical and optical sensing. LDHs used in sensor applications require large surface areas for analyte adsorption, high crystallinity for improved electrocatalytic activity, and excellent hosting ability for various intercalants. The electrocatalytic properties of LDHs enable the sensitive detection of an analyte as it undergoes a redox reaction. LDHs can also host various molecules with optical properties for fluorescent, colorimetric, or CL detection. Herein, standard methods of LDH characterization are overviewed using an exemplary study. Furthermore, the various electroanalytical and opto-analytical techniques are discussed.

### 3.1. LDH Characterization

LDH morphology, particle size, crystallinity, composition, and surface area are crucial to understanding and optimizing the performance of LDH-based sensors. Scanning electron microscopy (SEM) and transmission electron microscopy (TEM) are frequently used to characterize the shape, particle size, and interlayer spacing of the synthesized LDHs. X-ray diffraction (XRD) determines the degree of crystallinity and type of crystal structure present in the LDHs. Various spectroscopic methods such as X-ray photoelectron spectroscopy (XPS), Fourier-transform infrared spectroscopy (FT-IR), and energy-dispersive X-ray spectroscopy (EDX) elucidate the chemical composition of LDHs. The Brunauer–Emmett–Teller (BET) method characterizes the surface area and porosity of LDHs, which is especially important for gas-sensing LDHs [102,103,104].

A recent study by Li et al. exemplifies how the aforementioned characterization methods help optimize the performance of LDH-based nitrobenzene (NB) sensors [105]. Sodium dodecyl sulfate (SDS)-functionalized NiFe-LDHs NiFe–SDS-LDHs were synthesized via a hydrothermal reaction between NiCl_2_·6H_2_O, FeCl_3_·6H_2_O, CON_2_H_4_, NH_4_F, and SDS in a Teflon-lined autoclave. SEM and TEM found that the NiFe–SDS-LDHs with 250 mg SDS loading which exhibited a flocculent morphology comprising thin LDH nanosheets and a 4 μm particle size. High-resolution TEM (HRTEM) determined a lattice spacing of 0.231 nm for the NiFe-LDHs, which are characteristic of NiFe–CO_3_-intercalated LDHs, but found a larger 0.260 nm spacing for the NiFe–SDS-LDHs. The XRD of the NiFe–SDS-LDHs exhibited the same diffraction pattern as the NiFe–CO_3_-LDHs but with reduced peak intensities, suggesting that the SDS functionalization did not alter the crystal structure of the NiFe–CO_3_-LDHs but decreased the crystallinity. EDS elemental mapping determined a homogeneous distribution of Ni, Fe, and S atoms, with increasing S atoms at higher SDS loadings. FT-IR was found at characteristic peaks for –CH_2_ and S=O, further confirming the SDS intercalation. SDS functionalization was further confirmed via XPS, wherein the –OH/O_total_ ratio decreased for NiFe–SDS-LDHs than NiFe–CO_3_-LDHs. The similar Fe 2p and Ni 2p peaks suggested that the SDS did not affect the octahedral crystal structure. Lastly, BET isotherms determined that the NiFe–SDS-LDHs had a reduced surface area of 9.83 m^2^ g^−1^ than pristine NiFe-LDHs at 40.25 m^2^ g^−1^, which was expected because of the highly porous nanosphere structure in NiFe–CO_3_-LDHs. Although smaller BET surface areas theoretically hinder analyte adsorption, decreasing the LDH surface area minimized the hydrophilicity of LDHs. SDS functionalization with its long methylene chains further increased the hydrophobic character of the LDHs, which was crucial for attracting hydrophobic NB. Thus, the LDH sensor could be optimized by decreasing the surface area and maximizing SDS hydrophobicity. Studying the structural and chemical characteristics of LDHs allows for a deeper understanding of LDH sensor performance that may seem counter intuitive.

### 3.2. Electrochemical Detection

An electrochemical LDH sensor detects a change in the system’s electrical current due to the reversible redox reaction of an analyte [106]. LDH-based electrochemical sensors are often fabricated by depositing a thin film of catalytic LDHs onto substrates such as a GCE. [107]. Alternatively, LDHs can be directly grown on conductive substrates via an in situ method, yielding an electrochemical probe. The LDH-based probe is then exposed to various analytes in a three-electrode configuration and analyzed with different electroanalytical methods, including cyclic voltammetry (CV), chronoamperometry, differential pulse voltammetry (DPV), or square wave voltammetry (SWV), to characterize the LDH’s catalytic performance. The difference in the response current when exposed to different analyte concentrations is calibrated to determine the analyte concentrations in random samples.

CV is frequently used to characterize an LDH-based probe’s electrochemical properties. Tcheumi et al. utilized CV with [Fe(CN)_6_]^3−^ redox probe ions to measure the electrocatalytic ability of a NiAl-LDH sensor for isoproturon detection [108]. Highly crystalline NiAl-LDHs were synthesized via the co-precipitation of Ni(NO_3_)_2_·6H_2_O and Al(NO_3_)_3_·9H_2_O. An LDH-modified carbon paste was transferred to a Teflon tube to act as a NiAl-LDH/carbon electrochemical probe. The electrochemical properties of the LDH-modified carbon paste electrode (CPE) were studied via CV with [Fe(CN)_6_]^3−^ redox probe ions. Although the initial CV scan indicated a poor response current due to the low conductivity of the LDH, the LDH-modified probe exhibited a 49-fold higher response current after 40 cycles of multisweep CV. The increase in peak oxidation current was caused by increased [Fe(CN)_6_]^3−^ adsorption following each sweep. CV curves at different scan rates indicated that the active electrode area for the LDH-modified CPE (0.055 cm^2^) was higher than the bare CPE (0.047 cm^2^) owing to the high surface area of the LDH. The NiAl-LDH significantly increased the number of active sites for electrooxidation, resulting in higher response currents with increasing LDH content. However, due to the poor conductivity of the LDH, LDH loadings above 10 wt% decreased the sensor’s electrochemical response. When exposed to isoproturon, the NiAl-LDH-modified CPE exhibited a 2.6-fold higher response current than the pristine CPE due to the abundant isoproturon intercalation into the NiAl-LDH layers. SWV on five identically fabricated NiAl-LDH/CPEs indicated excellent reproducibility, indicated by a low 2% coefficient of variation for the response current. The peak oxidation current increased with higher analyte accumulation time up to 150 s, suggesting slow isoproturon intercalation. The LDH-based sensor retained 95% of its response current after 5 days, indicating excellent stability. SWV was also used to calibrate a linear detection range for isoproturon between 0.02 and 0.18 μM, with a low 1 nM LOD. A standard addition assay on spring water resulted in an excellent 97.5% recovery.

Chronoamperometry is a sensitive electroanalytical technique frequently used to characterize the performance of LDH-based sensors and calibrate the linear detection curve. Sahoo et al. fabricated a CoNi_2_-LDH for electrochemical DA sensing [41]. Bulk CoNi_2_-LDH was first synthesized via a hydrothermal process. The CoNi_2_-LDH had a highly crystalline rhombohedral structure with an average thickness of 4–6 nm (5–8 LDH layers) intercalated with water and CO_3_^2−^. Density functional theory (DFT) calculations on monolayer CoNi_2_-LDHs found that the adsorption of DA via van der Waals interactions was thermodynamically favorable. The Co atoms in the LDH lattice facilitated the charge transfer interactions between the LDHs and DA during electrooxidation. The exfoliated CoNi_2_-LDH was drop-cast onto a GC electrode with a Nafion binder. The LDH-modified electrode was used as the working electrode in a three-electrode configuration with a saturated calomel electrode (SCE) reference electrode and a Pt counter electrode in a 0.1 M phosphate-buffered solution (PBS).

**Figure 2:**
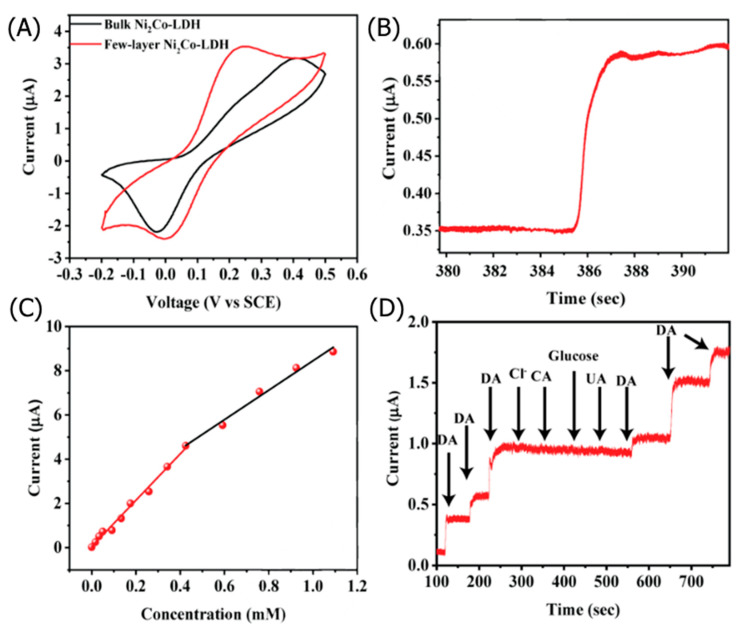
(**A**) CV curves for bulk and exfoliated CoNi_2_-LDH with 0.17 mM of DA at a scan rate of 50 mV s^−1^; (**B**) chronoamperometric response of exfoliated CoNi_2_-LDH; (**C**) calibration curve for the linear response of current vs. DA concentration; and (**D**) selectivity for DA sensing, reproduced from reference [41], with permission from Elsevier, 2021.

CV curves in Figure 2A indicated that the exfoliated CoNi_2_-LDH exhibited a lower oxidation peak potential at 0.24 V than bulk CoNi_2_-LDH (0.41 V), owing to the exfoliated LDH’s improved electron mobility. The cathodic peak current linearly increased with the square root of the scan rate, indicating a diffusion-dependent DA redox mechanism. Chronoamperometric analysis was performed at a 0.24 V oxidation potential, wherein the CoNi_2_-LDH exhibited a sharp increase in current when exposed to DA (Figure 2B). Two distinct linear detection ranges were calibrated for DA detection: 0.001–0.42 mM and 0.42–1.09 mM (Figure 2C). The LDH-based sensor exhibited a high sensitivity of 148.2 μA mM^−1^ cm^−2^ and a 3 s response time for DA concentrations above 0.42 mM. In addition to facile curve calibration and sensitivity, chronoamperometry allows facile interference studies, as presented in Figure 2D. The CoNi_2_-LDH did not significantly respond to interfering glucose, Cl^−^, uric acid (UA), and citric acid (CA), whereas DA exposure caused a large spike in the response current. Thus, chronoamperometry determined that the LDH-based electrode exhibited excellent selectivity for DA.

DPV is another sensitive electroanalysis technique frequently used to determine the analytical performance of LDH-based sensors. Qiao et al. utilized DPV to evaluate a CdAl-LDH-based anthracene sensor [109]. A CdAl-LDH-modified probe was synthesized via in situ electrodeposition of Cd(NO_3_)_2_·6H_2_O and Al(NO_3_)_3_·9H_2_O onto a GCE. Vertically aligned CdAl-LDH nanoflakes were homogeneously grown on the surface of the GCE, resulting in a thin-film LDH coating. Probes with increased LDH film thickness exhibited higher electron transfer resistances, owing to an increase in insulative LDH nanosheets. The LDH-modified electrode was used as the working electrode in a three-electrode configuration with an Ag/AgCl reference electrode and Pt counter electrode in a 0.1 M KOH electrolyte. CV curves indicated a 43% decrease in the oxidation response current when exposed to anthracene. OH^−^ intercalation played a critical role in reducing Cd^2+^ to Cd; however, the hydrophobicity of anthracene inhibited OH^−^ diffusion. Thus, increasing anthracene concentrations decreased the LDH’s catalytic activity. The anodic response current linearly increased with the CV scan rate, indicating a surface-controlled redox mechanism for Cd reduction. Because DPV provides greater analytical sensitivity than CV, DPV was used to calibrate the linear range for anthracene [109]. The linear detection range was 0.1–100 pM with an incredibly low LOD of 0.5 fM. Other polycyclic aromatic hydrocarbons such as naphthalene, phenanthrene, benzene, benzo[*a*]pyrene, and benzo[*a*]anthracene decreased the peak current by <20%, whereas anthracene decreased the response current by 43%. The CdAl-LDH demonstrated excellent probe stability with a 97% response current retention after 30 days in 4 °C refrigeration. Five identical LDH-based sensors exhibited a 5.3% relative standard deviation (RSD), suggesting good reproducibility. Standard addition assays on cloud water and rainwater from Mount Taishan found an overestimation of anthracene content due to the presence of other polycyclic aromatic hydrocarbons. However, the recoveries were still high at 98.7% and 99.1% for cloud water and rainwater, respectively.

Electrochemical analysis techniques, such as CV, are insightful methods for characterizing LDH-based electrochemical probes. More sensitive techniques, including chronoamperometry and DPV, enable the fast and accurate calibration of analyte concentrations for applications in real-world samples. Chronoamperometry, in particular, provides a simple method of determining LDH sensor selectivity.

### 3.3. Optical Detection

Optical detection relies on measuring changes in a sensor’s fluorescence, color, or chemiluminescence in response to various analytes. LDHs are utilized as versatile host materials that immobilize various light-interacting compounds, wherein the change in absorbance or emission is detected when exposed to analytes. LDHs can also be composed of spectroscopic materials during electrode fabrication for similar opto-analysis. Alternatively, LDHs can catalyze chemiluminescent (CL) or color-changing reactions, such as H_2_O_2_-TMB, when exposed to different analytes.

Fluorescence spectroscopy is one of the most common opto-analytical techniques used to determine the concentration of various analytes using fluorophore-doped LDH sensors. Zhang et al. fabricated a fluorescent sensor for the bovine serum albumin protein by intercalating fluorescent 8-anilino-1-naphthalenesulfonate (ANS) dye into a MgAl–NO_3_-LDH [110]. The MgAl–NO_3_-LDH was first synthesized via a co-precipitation reaction in a mechanochemical process under a nitrogen atmosphere. An acid-treated quartz substrate was alternatingly dipped into either the LDH suspension or ANS solution, yielding a thin-film LDH coating with ANS immobilized between MgAl-LDH nanosheets. The surface roughness of the thin film increased with additional bilayers. The negatively charged sulfonate groups on the ANS exhibited a high affinity for the positively charged brucite-like layers of the LDH. Moreover, the imino groups on the ANS formed hydrogen bonds with the LDH, resulting in the strong adsorption into the lamellar LDH structure. UV–Vis spectroscopy and fluorescence spectroscopy were conducted to determine the optical properties and opto-analytical potential of the LDH. Increasing the number of 7.2 nm-thick MgAl-LDH/ANS bilayers to a maximum of 20 bilayers increased the UV–Vis absorbance—owing to increased ANS content. The LDH-modified probe exhibited a broad emission peak centered at 468 nm. When exposed to the bovine serum albumin protein, the fluorescence intensity increased due to structural changes in the ANS via intermolecular interactions with the albumin protein. Furthermore, the fluorescence intensity increased in non-polar solvents due to weaker interactions between the ANS and solvent. UV–Vis absorbance was used to calibrate the concentration of the albumin protein. The LDH sensor with 10 bilayers exhibited a 1.37 mg L^−1^ detection limit for the albumin protein with two linear detection ranges: 0.02–0.12 g L^−1^ and 0.12–0.28 g L^−1^. The sensor demonstrated high selectivity for the albumin protein, indicated by minimal interference from common biomolecules such as glucose, glutathione, L-cysteine, and pancrelipase. LDHs have favorable guest–host interactions with various fluorophores for effective fluorometric analyte detection.

**Figure 3:**
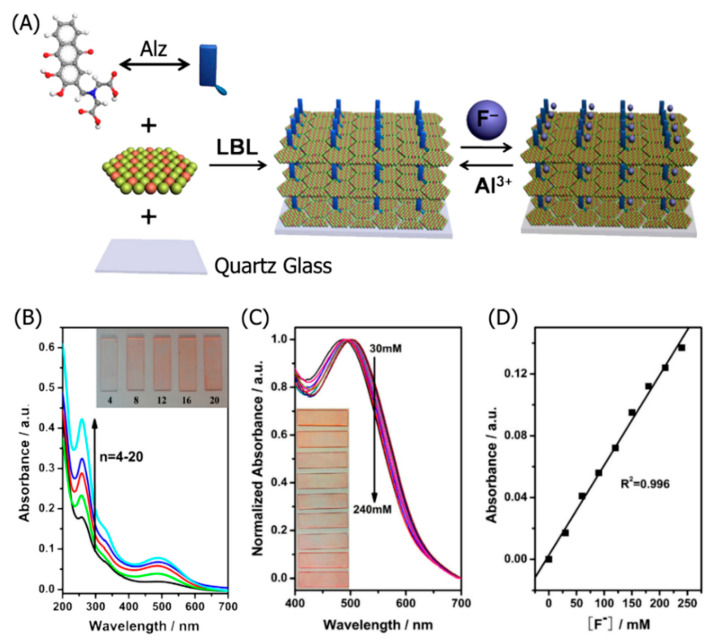
(**A**) Schematic of Mg_2_Al-LDH/Alz optical sensor fabrication and F^−^ adsorption for optical detection; (**B**) change in absorbance for the LDH/Alz sensor with an increasing number of bilayers from 4 to 20; (**C**) bathochromic shift in peak absorbance with increasing F^−^ concentration from 30 to 240 nM; and (**D**) the linear calibration curve of F^−^ concentration based on the change in absorbance at 550 nm for as a function of F^−^ concentration for the MgAl_2_-LDH/Alz sensor with 20 layers, reproduced from reference [111] with permission from Elsevier, 2013.

The colorimetric analysis method is another common technique used to determine the concentration of various analytes. A major advantage of the colorimetric technique is its instantaneous “naked eye” detection. Jia et al. fabricated a colorimetric Mg_2_Al-LDH/alizarin complexone (Alz) sensor to detect F^−^ [111]. Mg_2_Al-LDH nanoparticles were first synthesized via a hydrothermal process. Alternating dip-coating of a quartz glass substrate into a Mg_2_Al-LDH dispersion and Alz solution yielded a lamellar, thin-film optical probe (Figure 3A). A single Mg_2_Al-LDH/Alz bilayer was 6.28 nm thick, resulting in a 138 nm-thick film with 20 bilayers. Increasing from 8 to 20 bilayers resulted in increased absorbance, owing to increased Alz content and a more homogeneous thin-film coating (Figure 3B). UV–Vis spectroscopy was conducted using a spectrophotometer with a 4.0 nm slit within a range of 200–700 nm. Adsorption of F^−^ into the Mg_2_Al-LDH/Alz composite caused a bathochromic shift from 486 to 504 nm, observed as a color change from orange to pink (Figure 3C). The colorimetric detection method enabled the naked-eye detection of F^−^. The changes in the peak absorbance at different F^−^ concentrations were calibrated, yielding a 30–250 μM linear detection range with a 12.9 μM LOD (Figure 3D). The LDH-based colorimetric probe exhibited complete reversibility upon washing with an Al^3+^ solution, with a 1 s recovery time, even after 10 cycles. The immobilization of Alz in the LDH layers prevented Alz aggregation and improved its stability, indicated by the high 95% absorbance retention after 1 month. The LDH-based sensor exhibited excellent selectivity, indicated by low interference from common anions, such as Cl^−^, Br^−^, NO_3_^−^, and HSO_4_^−^. Standard addition assays using tap and lake water found high recoveries between 96.51% and 107.06%. LDHs can be used for the fast colorimetric detection of various analytes.

CL analysis takes advantage of the excellent catalytic ability of LDHs to enhance emissions from various CL systems. Xie et al. used CoFe-LDH nanoparticles to catalyze the H_2_O_2_–luminol CL reaction system for effective H_2_O_2_ detection [112]. CoFe-LDH was synthesized via a hydrothermal co-precipitation process. The resulting CoFe-LDH comprised thin, circular nanoplates, which afforded many active sites to catalyze the H_2_O_2_–luminol reaction system. The CoFe-LDH was injected and carried by a stream of water into a mixing tube, wherein the LDH was exposed to the luminol/H_2_O_2_ solution. The CoFe-LDHs increased the CL intensity of the H_2_O_2_–luminol reaction by 170 times, owing to the peroxidase-like catalytic activity of the LDHs. Increasing the pH of the buffer solution to 10.98 yielded the maximum CL intensity, peaking at a wavelength of 425 nm. A 5 μM luminol concentration exhibited the highest S/N ratio. The CoFe-based H_2_O_2_ sensor exhibited a linear detection range of 0.01–2 μM and a low 5 nM detection limit. The CoFe-LDH-based sensor exhibited strong selectivity against various metal cations and polyatomic anions with recoveries >95%. Certain metal cations, such as Fe^3+^ and Co^2+^, caused more significant interference, which could be mitigated by adding ethylenediaminetetraacetic acid as a chelating agent. A standard addition assay found recoveries between 85% and 107.5%, with an RSD of 2.1% for 11 rainwater samples.

Optical analyte detection is fast and sometimes does not require expensive analysis tools. Measuring changes in the absorbance, fluorescence, or chemiluminescence of LDH-based probes when exposed to different analytes enables the fine calibration of analyte content. Moreover, colorimetric methods enable naked-eye detection for instant detection of harmful substances.

## 4. Analyte Detection

Sensors play a critical role in monitoring human health and environmental safety. Unusually high or low levels of biological molecules, such as glucose and dopamine, can cause severe damage to the human body. High concentrations of pollutants, such as H_2_O_2_, nitrogen-based toxins, and metal ions, harm humans and the environment. Various LDH-based sensors have been developed as fast, affordable, and accurate sensors for many critical chemical markers. Herein, the design and modifications of various LDHs and LDH composites are examined, emphasizing the effects of LDH morphology, crystallinity, and composition on analytical performance.

### 4.1. Glucose

The ability to quickly and accurately monitor glucose levels in the blood is critical for diagnosing, managing, and treating diabetes [113]. If left unchecked, people with diabetes may experience fatal kidney failures, blindness, heart attacks, and strokes [114]. Electrochemical glucose sensors detect electron flow from glucose oxidation into gluconolactone, promoted by a catalyst, such as an enzyme, noble metal, transition metals, or metal oxides [115]. Various LDHs have been fabricated as catalysts for electrochemical and optical glucose detection.

**Figure 4:**
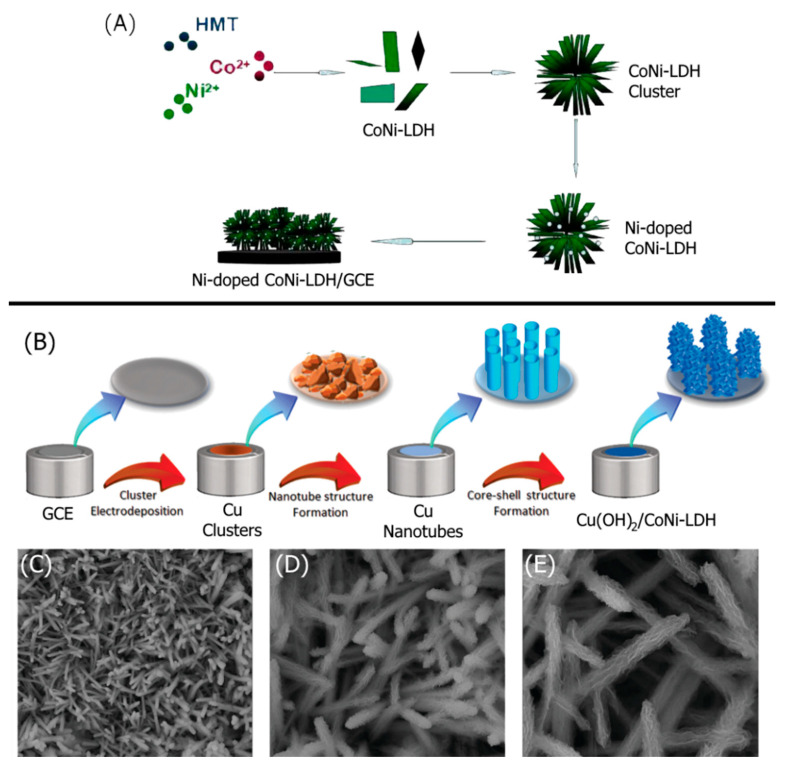
Schematic illustration of the fabrication of (**A**) Ni-doped CoNi-LDH on GCE, reproduced from reference [116], with permission from Wiley, 2016 and (**B**) Cu(OH)_2_/CoNi-LDH; (**C**–**E**) SEM micrographs of the Cu(OH)_2_/CoNi-LDH at increasing magnifications, reproduced from reference [48] with permission from The Royal Society of Chemistry, 2019.

One of the largest disadvantages of fabricating LDH-based glucose sensors is the reduced conductivity due to the insulative property of LDHs and the binders used to immobilize the LDHs onto the conductive substrate. One method of improving the electrochemical glucose detection mechanism is to enhance conductivity by adding conductive nanoparticles, such as Ni nanoparticles, to the LDHs. LDHs can be partially reduced to yield free metal nanoparticles that reduce the impedance of the electrochemical probe. CoNi-LDHs have received much attention as efficient catalysts for glucose electrooxidation, owing to the similar potentials between Co and Ni that synergize to increase the composition of their electronic states. However, CoNi-LDHs exhibit poor conductivity that can be improved by LDHs comprised of conductive nanomaterials. Chen et al. extracted Ni nanoparticles by partially reducing CoNi-LDHs to yield a nanocomposite with enhanced conductivity for improved glucose detection [116]. The CoNi-LDH was first synthesized via a hydrothermal method with Ni(NO_3_)_2_·6H_2_O, Co(NO_3_)_2_·6H_2_O, and hexamethylenetetramine (HMT). The CoNi-LDH underwent partial hydrothermal reduction with Na_2_HPO_4_ and NaOH at 160 °C for 4 h to yield free Ni nanoparticles from the CoNi-LDHs (Figure 4A). The LDHs comprised large nanoflowers formed by interconnected nanosheets with 10–20 nm thickness, 200 nm lateral length, and tiny intercalated Ni nanospheres. A suspension of the CoNi-LDH/Ni nanocomposite was drop-cast onto a polished GCE with a chitosan binder, yielding an LDH-based glucose probe. The application of a chitosan binder and insulative CoNi-LDHs on the conductive GCE increased electrical impedance. However, the free Ni nanoparticles in the LDH-based film decreased the electron transfer resistance and facilitated glucose electrooxidation, owing to the high conductivity of the Ni nanoparticles and their ability to act as active sites for glucose electrooxidation. Thus, the CoNi-LDH/Ni composite exhibited a higher response current than the bare CoNi-LDH. Chronoamperometry found that the CoNi-LDH/Ni composite sensor exhibited excellent analytical ability, indicated by the low LOD of 1.6 μM and two wide linear detection ranges: 0.005–1.2 and 1.2–14.8 mM. Commonly interfering biomolecules such as DA, uric acid (UA), and ascorbic acid (AA) had no significant effect on glucose detection. A standard addition assay on blood samples exhibited a good recovery range between 97.3% and 101.5%. LDHs that are already excellent catalysts for glucose electrooxidation can be easily enhanced by extracting some of their metal centers via partial reduction to yield conductive nanoparticles adsorbed into the LDH nanosheets for improved conductivity.

Another way to reduce electrical impedance for more sensitive electrochemical glucose detection is to directly synthesize LDHs on conductive substrates to avoid using an insulative binder material. While it is challenging to directly synthesize LDHs due to poor adhesion between the lamellar LDHs and smooth metal substrates, CoNi-LDHs have been successfully grown on conductive electrodes using conductive nanotubes as nucleation points for LDH crystal growth. Shahrokhian et al. used vertical Cu(OH)_2_ nanotubes stably grown on a GCE as a template on which to synthesize the CoNi-LDHs [48]. The composition with Cu(OH)_2_ nanotubes removed the need for a binder and improved the conductivity of the electrode as electrons moved freely along the length of the Cu(OH)_2_ nanotubes. A GCE was first electrodeposited with a layer of Cu that was subsequently oxidized into Cu(OH)_2_ nanotubes with (NH_4_)_2_S_2_O_8_ (Figure 4B). CoNi-LDH sheets were directly grown on the Cu(OH)_2_-coated GCE via electrodeposition, using the Cu(OH)_2_ nanotubes as essential nucleation sites. The hydroxyl groups of Cu(OH)_2_ attracted Co^2+^ and Ni^2+^ for homogeneous LDH nucleation. The resulting Cu(OH)_2_/CoNi-LDH core/shell nanostructure is shown in Figure 4C–E. The Cu(OH)_2_ nanotubes, which had a 50–250 nm diameter and 2–3 μm length, provided a hollow nanostructure that promoted ion intercalation and access to additional active sites. Moreover, the direct adhesion between CoNi-LDHs and Cu(OH)_2_ improved the electrical conductivity, owing to the absence of an insulative binder material. Electrodeposition durations of 60 s resulted in loosely formed LDH layers, whereas depositions longer than 75 s resulted in unproductive LDH agglomerations. Seventy-five seconds of LDH deposition resulted in a porous LDH shell with a 50–100 nm thickness.

Chronoamperometric analysis of the CoNi-LDH/Cu(OH)_2_/GCE sensor determined a low glucose detection limit of 0.6 μM at a signal/noise ratio (S/N) of 3 and two linear ranges: 0.002–3.2 mM and 3.2–7.7 mM. The sensitivity decreased from 1895 to 1322 μA mM^−1^ cm^−2^ at higher glucose concentrations, owing to more gluconolactone aggregations on the LDH surface at higher glucose concentrations that inhibited additional glucose adsorption [48]. The binder-less construction and highly porous architecture enabled fast electron and ion mobility, increasing the oxidation current response. The CoNi-LDH/Cu(OH)_2_/GCE sensor exhibited excellent anti-interference from other biomolecules and saccharides, such as DA, UA, AA fructose, sucrose, and lactose. The binder-less LDH glucose sensor demonstrated good stability, indicated by the 6.37% RSD for five repeated glucose measurements. A standard addition test using human blood samples exhibited recoveries between 103.5% and 108.6%. Thus, the direct growth of catalytic CoNi-LDHs on conductive substrates is an effective means of improving electrochemical glucose detection.

While the direct synthesis of CoNi-LDHs on conductive substrates using conductive nanotubes is advantageous by virtue of avoiding the insulative binder material, the advantages of the conductive nanotube/LDH core/shell nanostructures must be emphasized. Zhao et al. improved the conductivity of a CoNi-LDH-based sensor via the in situ growth of the LDH on a conductive Cu foam (CuF) substrate modified with cobalt copper carbonate hydroxide (CCCH) nanorods as nucleation points [47]. The resulting CCCH/CoNi-LDH core/shell nanostructure possessed high surface area and conductivity for enhanced electrochemical glucose detection. A hydrothermal method was first used to coat CuF with homogeneously protruding, needle-like cobalt copper carbonate hydroxide (CCCH) nanorods. CoNi-LDH nanosheets were grown on the CCCH nanorods via a one-pot hydrothermal process via a reaction between CoCl_2_·6H_2_O, Ni(NO_3_)_2_·6H_2_O, hexadecyl trimethyl ammonium bromide, and the CCCH/CuF substrate. The conductive CCCH/CuF template provided a porous microstructure and many nucleation points for homogeneous CoNi-LDH growth. While the core/shell nanostructure already provided a high surface-area template, the nanocomposite structure was optimized for maximum surface area by tuning the Co-to-Ni ratio. An LDH with a low Co content resulted in non-uniformly sized bulk nanostructures with a ~100 nm layer thickness. Increasing the Co ratio to 4:6 (Ni to Co) improved the alignment of the LDH layer with the CCCH nanorods, yielding more uniform nanosheets and decreased LDH layer thickness for more abundant active sites. Further increasing the Co content shifted the crystal structure from an LDH phase to a Co_2_(OH)_3_Cl phase, reducing the response current. The growth of the optimized CoNi-LDH nanosheets on the CCCH/CuF electrode increased the surface area by 1.85 times. The hydrothermal reaction duration also significantly influenced the growth of CoNi-LDHs, with reactions under 10 h yielding only tiny nanosheets. A 10 h crystallization period formed sufficient CoNi-LDH layers without significant aggregations, whereas longer durations resulted in neighboring LDHs connecting and decreasing the overall microporosity. The highly porous structure enabled fast ion diffusion and more active sites for glucose adsorption and electrooxidation. The highly conductive CCCF/CuF also facilitated electron transfer for improved redox kinetics. As such, chronoamperometry found that the optimized CoNi-LDH with a Co-to-Ni ratio of 4:6 exhibited a linear detection range of 0.001–1.5 mM, a high sensitivity of 10780 μA mM^−1^ cm^−2^, a low LOD of 0.68 μM, and a short recovery time of 2.4 s. Weekly use of the sensor over 42 days resulted in no significant change to the response current. The CoNi-LDH did not experience significant interference from common biomolecules, such as UA, citric acid, and fructose. The sensor exhibited excellent recoveries between 98.5% and 102.6% when tested on human serum samples via the standard addition method. CoNi-LDHs can be directly synthesized onto conductive substrates using stable nanotubes as nucleation points, yielding binder-less glucose probes. However, the performance of CoNi-LDH sensors can be further improved by optimizing their morphology for maximum surface area and electron mobility by tuning the metal composition and LDH growth duration.

**Figure 5:**
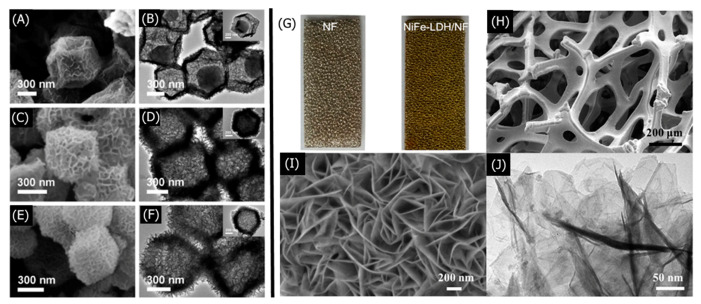
SEM and TEM micrographs of (**A**,**B**) Co_0.52_Ni_0.48_-LDH; (**C**,**D**) Co_0.33_Ni_0.67_; and (**E**,**F**) Co_0.21_Ni_0.79_ reproduced from reference [117] with per mission from American Chemical Society, 2019 (**G**) Digital photograph of Ni foam and NiFe-LDH modified Ni foam. (**H**) Low and (**I**) high magnification SEM micrographs of NiFe-LDH on Ni Foam. (**J**) TEM of the NiFe-LDH nanosheets reproduced from reference [46] with permission from Wiley, 2017.

The optimization of the Co-to-Ni ratio is vital for yielding highly catalytic LDHs with a porous structure. Kong et al. used a metal–organic framework (MOF) template and varied the Co-to-Ni ratio to yield hollow shell Co_x_Ni_1-x_-LDHs with different nanostructures [117]. Ni(NO_3_)_2_·6H_2_O was added to a dispersion of ZIF-67 in ethanol, yielding thin nanosheets on the surface of the hollow ZIF-67. The template-synthesized Co_0.52_Ni_0.48_-LDH retained the original dodecahedral shape of the ZIF-67 MOF, resulting in a ZIF-67/LDH yolk/shell structure (Figure 5A,B). Increasing the Ni content to Co_0.33_Ni_0.67_ and Co_0.21_Ni_0.79_ resulted in no yolk due to the growth of more CoNi-LDH nanosheets (Figure 5C–F). Higher Ni compositions resulted in a higher BET surface area, increasing from 269 m^2^ g^−1^ (Co_0.33_Ni_0.67_) to 358 m^2^ g^−1^ (Co_0.21_Ni_0.79_), owing to the formation of denser LDH nanosheets with increased Ni content. Compared to a pure Co-LDH-based probe, the CoNi-LDH-based probe exhibited an enhanced catalytic activity, owing to its more porous architecture for abundant active sites and efficient ion diffusion. Sufficient Co content was essential in yielding maximum electrocatalytic activity as demonstrated by the decreased analytical ability of the Co_0.21_Ni_0.79_-LDH probe rather than the Co_0.33_Ni_0.67_-LDH probe despite it having a larger surface area and Ni content. Thus, the Co centers were primarily responsible for the glucose electrooxidation mechanism, whereas the Ni content improved LDH formation while contributing some electrooxidation. Chronoamperometric analysis found a linear detection range from 0.01 to 2 mM with a sensitivity of 242.9 μA mM^−1^ cm^−2^ and an LOD of 3.1 μM. The MOF-based CoNi-LDH exhibited excellent anti-interference against common biomolecules such as AA, DA, and UA, as well as other saccharides such as sucrose, fructose, and lactose. The CoNi-LDH sensor was also highly stable, as indicated by the 91.7% response retention after 7-day exposure to air. When dealing with CoNi-LDHs, the metal ratios must be tuned to yield LDHs with a high surface area and electrocatalytic activity.

The ratio between more dissimilar metals, such as Ni and Fe, may be less impactful to the overall structure of the LDHs, resulting in less influence on electrochemical glucose sensing. Instead, improving the conductivity of Ni-based LDHs is more crucial for enhanced detection, owing to the innate electrocatalytic ability of Ni. Moolayadukkam et al. found no significant morphological nor crystallinity changes of a NiFe-LDH when altering the Ni-to-Fe ratio from 2:1 to 4:1 [118]. The Ni_x_Fe_1−x_-LDHs were synthesized via urea hydrolysis. Each LDH exhibited a rhombohedral LDH crystal phase and no significant changes to the lateral length of the LDH platelets. While the morphology of the NiFe-LDH did not change, the selectivity for glucose electrooxidation against the oxygen evolution reactions (OERs) was optimized with a Ni-to-Fe ratio of 4:1, indicated by the widest separation between the glucose oxidation peak and OER peak. DFT simulations revealed that increasing Ni content raised the required hydrogen desorption energy that inhibited the OER. The various NiFe-LDHs were composited with rGO for improved conductivity without changing the morphology. Ni_4_Fe-LDH/rGO_5_ (5 wt% rGO) produced the maximum peak response current, owing to the improved conductivity and increased surface area provided by the rGO. However, further increasing rGO content reduced the response current due to less NiFe-induced glucose electrooxidation. The chronoamperometric analysis of the optimized LDH/rGO sensor determined a sensitivity of 176.8 μA mM^−1^ cm^−2^ in a linear detection range of 0–3.1 mM. The sensor also operated without interference from UA and Cl^−^. Improving LDH conductivity via doping with conductive rGO may be more valuable than optimizing the Ni-to-Fe ratios in NiFe-LDHs.

The conductivity of NiFe-LDHs can also be enhanced by directly synthesizing the LDHs onto conductive substrates. In this case, NiFe-LDH morphologies must be optimized for maximum glucose adsorption. Lu et al. synthesized a NiFe-LDH-based glucose sensor via the in situ growth of NiFe-LDHs on Ni foam using a hydrothermal process [46]. The growth of the NiFe-LDHs changed the color of the Ni foam from silver to bronze (Figure 5G). The NiFe/Ni foam probe comprised vertically aligned NiFe-LDH nanosheets (Figure 5I) homogeneously grown on the Ni foam template with minimal aggregations (Figure 5J). While the exact Ni-to-Fe ratio may not drastically influence the structure, the Fe centers were crucial in reducing Ni self-aggregation. Thus, adequate Fe^3+^ content was necessary for high porosity with pore sizes greater than 50 nm (Figure 5H), enabling rapid ion diffusion and abundant active sites. Unlike in the previously analyzed CoNi-LDHs [117], the Ni centers facilitated glucose electrooxidation, while the Fe centers optimized the structure. Chronoamperometry determined a wide 2–800 μM linear detection range with a high sensitivity of 3680.2 μA mM^−1^ cm^−2^ and an LOD of 0.59 μM. The sensor exhibited high selectivity against interfering biomolecules such as DA and lactose, even at a 1:1 ratio with glucose. The binder-less electrode demonstrated excellent stability and reproducibility, exemplified by a 98% sensitivity retention after 45 days and a low RSD of 5.37% between five identical electrodes. Standard addition assays on human serum samples found high 95.6–98% recoveries. Binder-less Ni-based LDH glucose sensors can exhibit enhanced electroanalytical performance and stability.

NiAl-LDHs benefit from the catalytic Ni metal centers synergizing with the highly conductive and lightweight Al^3+^ sites. However, NiAl-LDHs exhibit high electrical impedance when deposited on conductive probes, reducing glucose detection sensitivity. The same methods of improving the conductivity for CoNi-LDHs and NiFe-LDHs can be applied to NiAl-LDHs. A common method of increasing NiAl-LDH conductivity is to compose the LDHs of conductive metals and carbon nanoparticles. Fu et al. improved the conductivity of NiAl-LDHs by doping Au nanoparticles in a NiAl-LDH and having Au-doped/LDHs comprised of CNT/graphene oxide (GO) [119]. The NiAl-LDH/carbon nanocomposite was first synthesized via in situ co-precipitation, wherein the appropriate metal nitrate solutions were mixed with GO and CNT followed by precipitation with NaOH. The NaOH used during the co-precipitation process also reduced the GO to rGO, yielding more graphene-like characteristics for higher conductivity. The resulting LDHs were mixed with HAuCl_4_ and polyvinylpyrrolidone, yielding an Au/LDH/CNT/rGO composite. The LDH-based composite comprised a 20–30 nm-long NiAl-LDH sheets homogeneously distributed on exfoliated rGO sheets. Long CNTs were interwoven between the large rGO sheets via π–π stacking. The LDH/carbon immobilization matrix hosted 0.5 wt% Au nanoparticles around 8.4 nm in diameter, resulting in a 3D hybrid material. The LDH-modified GCE exhibited a low charge transfer resistance of 2.25 Ω cm^2^, a higher conductivity than pristine CNTs and rGO, owing to improved carbon dispersion facilitated by the LDH. The lamellar NiAl-LDHs provided abundant active sites for glucose electrooxidation, while the Au nanoparticles and carbon matrix improved electron mobility for faster reaction kinetics. The Au nanoparticles also helped adsorb hydroxide anions, which were required for glucose oxidation. Chronoamperometric analysis of the LDH-based probe found a broad linear detection range of 0.010–6.1 mM, a high sensitivity of 1989.0 μA mM^−1^ cm^−2^, and an LOD of 1.0 μM. The 3D architecture of the LDH nanocomposite enabled the fast diffusion of glucose to the lamellar LDH. The Au/LDH/CNT/rGO sensor demonstrated high selectivity with less than 3% current response deviations against similar biomolecules and saccharides, including AA, DA, sucrose, and lactose. The electrode exhibited high stability with a 95% current retention after 30 days and a 4.1% RSD for five consecutive glucose measurements. The glucose sensor demonstrated excellent reproducibility, with a 1.9% RSD for five identically constructed electrodes. Standard addition assays on human blood samples found that the LDH-based glucose sensor exhibited excellent recovery values between 98.4% and 101.1%. Shishegari similarly improved the conductivity of NiAl-LDHs by compositing with Pd nanoparticles and nitrogen-doped rGOs (NrGOs) [120]. The NiAl-LDHs/Pd/NrGO composite was synthesized via the one-pot electrodeposition of PdCl_2_, Ni(NO_3_)_2_, Al(NO_3_)_3_, and KNO_3_ on a graphite substrate. Electrochemical characterization using [Fe(CN)_6_]^3−/4−^ redox probe ions found a higher response current for the LDH/NrGO electrode than the NrGO-less probe, owing to the increased surface area and conductivity afforded by the NrGO. The addition of Pd nanoparticles further enhanced the nanocomposite’s conductivity, indicated by the decrease in the charge transfer resistance from 3765 Ω to 2840 Ω without and with Pd nanoparticles, respectively. The intercalated Pd nanoparticles also enhanced the glucose electrooxidation peak current by facilitating OH^−^ adsorption. Chronoamperometry was used to determine the analytical performance of the hybrid LDH electrode. The Pd–NiAl–LDH/NrGO sensor exhibited a linear detection range of 0.5–10 000 μM, a sensitivity of 315.46 μA mM^−1^ cm^−2^, and a 234 nM detection limit. The probe demonstrated excellent anti-interference against AA, UA, DA, and other common biomolecules. The advantages of the doped metal nanoparticles and carbon nanomaterials go beyond simply enhancing LDH conductivity. Catalytic Ag and Pd nanoparticles showed the ability to increase OH^−^ adsorption, which was required for the glucose electrooxidation reaction mechanism. Similar metal nanoparticles with adsorptive properties may be implemented in LDH-based sensors for increased electrocatalysis. Carbon nanoparticles such as CNTs and rGO provide a porous template for LDHs to grow on, enabling increased glucose diffusion and more active sites for electrooxidation.

Apart from using the LDHs to directly catalyze glucose electrooxidation, LDHs are excellent host materials probe molecules. Various Co-based LDHs have been used as hosts for both electrochemical and optical detection. Wu et al. synthesized CoAl-LDHs hosting alizarin red S/aminophenylboronic acid complexes (ARS-PBA) for electrochemical glucose detection [121]. The CoAl-LDH was first produced via a hydrothermal reaction between Co(NO_3_)_2_·6H_2_O and Al(NO_3_)_3_·9H_2_O in NaOH. ITO glass was subsequently dip-coated with alternating layers of ARS-PBA and CoAl-LDH. The resulting probe comprised hexagonal plate-like microstructures with highly crystalline CoAl-LDHs. Ten alternating layers of 7.4 nm-thick CoAl-LDH/ARS-PBA bilayers exhibited the lowest electron transfer resistance of 26.71 Ω and the highest peak oxidation current. The CoAl-LDHs prevented ARS-PBA aggregation and increased the surface area for improved catalytic activity. The DPV of the LDH/ARS-PBA glucose sensor found a linear detection range of 0–1 μM and a low LOD of 4 nM. The LDH-based sensor demonstrated excellent selectivity against DA, UA, and AA. The sensor was also highly stable with minimal response current loss after 10 days, owing to the LDH inhibiting ARS-PBA from peeling off the ITO substrate. The excellent hosting ability of LDHs enables LDH-based sensors to adsorb catalytic molecules for electrochemical glucose detection. The role of the LDHs as hosts is to provide chemical and physical stability as well as increased surface area for improved catalysis.

**Figure 6:**
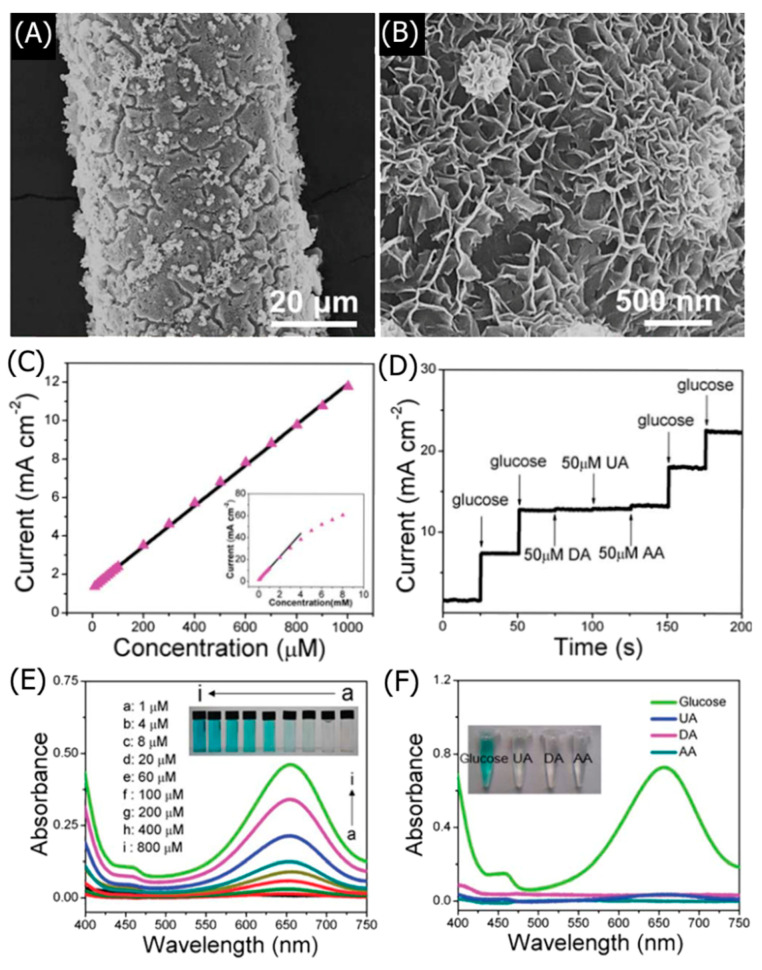
SEM micrographs of the CoFe-LDH at (**A**) low and (**B**) high magnifications; (**C**) calibration of the CoFe-LDH sensor for glucose detection for the linear portion and the complete calibration curves (insert); (**D**) chronoamperometry with glucose, DA, UA, and AA for interference studies. Change in absorbance with increasing glucose concentration from 1 to 800 μM (**a**–**i**). Optical selectivity for glucose against UA, DA, and AA (**E**) UV-vis spectra and photographs for the CoFe-LDH colorimetric system with different glucose concentrations; (**F**) Selectivity tests of the CoFe-LDH colorimetric system ([DA] = [AA] = [UA] = 5 mM, [glucose] = 2 mM), reproduced from reference [122] with permission from The Royal Society of Chemistry, 2019.

LDHs can perform simultaneous optical and electrochemical detection as the LDHs act as hosts for optical probe molecules while directly oxidizing glucose with its transition metal centers, such as Co^2+^. Cui et al. fabricated a simultaneous electrochemical and colorimetric glucose sensor based on a CoFe-LDH with adsorbed chromogenic TMB [122]. Electrodeposition of CoFe-LDHs onto a Ni wire yielded vertically aligned LDH nanosheets with ~8 nm thickness and 250–300 nm lateral length (Figure 6A,B). The Co metal centers oxidized glucose, which was detected as a change in peak current. While the Co in the CoFe-LDH contributed to most of the glucose electrooxidation, the Fe facilitated electron transfer by altering the coordination electron structure of the LDH. The chronoamperometric analysis of the LDH-based glucose sensor determined a linear detection range of 10–1000 μM, a detection limit of 0.27 μM, and a high sensitivity of 1.063 μA μM^−1^ cm^−2^ (Figure 6C). The sensor demonstrated high selectivity against UA, AA, and DA (Figure 6D). It also exhibited high cyclability, indicated by the 2.2% RSD for 10 cycles. The CoFe-LDH also oxidized TMB when exposed to glucose, converting the TMB from colorless to sky blue with a peak absorbance at a wavelength of 652 nm (Figure 6E).

The colorimetric aspect had a smaller linear detection range of 1–20 μM and a higher detection limit of 0.47 μM. The colorimetric sensor exhibited high selectivity against DA, AA, and UA and good stability with a 95% absorbance retention after three repeated tests (Figure 6F). Both the electrochemical and colorimetric analytical methods were successfully conducted on human urine samples, with both methods exhibiting the same linear detection range. By using LDHs as hosts and catalysts, sensors can benefit from accurate electrochemical detection and naked eye optical detection.

Various LDHs have been modified to improve their catalytic ability for electrochemical glucose detection. The performance of LDH-based glucose sensors is summarized in Table 1. Among the largest drawbacks of LDH-based sensors is their poor conductivity; conductive metal nanoparticles or carbon nanostructures may be added to enhance electron mobility for faster redox kinetics. Moreover, catalytic metal nanoparticles facilitate OH^−^ adsorption for faster glucose oxidation. Carbon nanostructures provide a porous template for increased diffusion rates and abundant active sites. Different LDH materials may be directly grown on conductive substrates for lower electron transfer resistance and enhanced stability. LDHs are also fantastic hosts for electrochemical or optical probe molecules. Combined with the natural glucose oxidation ability of Co and Ni-based LDHs, LDH sensors enable simultaneous electrochemical and optical detection for accurate and naked-eye detection.

### 4.2. Dopamine

DA is a vital neurotransmitter that affects critical human organ systems, including the cardiovascular, endocrine, and renal systems [123]. However, abnormal DA levels have been linked to various mental illnesses, including depression, schizophrenia, Alzheimer’s, and Parkinson’s disease [124]. Therefore, developing accurate and affordable DA sensors is imperative for preventing, diagnosing, and treating such diseases [125]. LDHs are promising materials for electrochemical and optical DA detection, with their low cost, high sensitivity, selectivity, and biocompatibility. However, the poor conductivity of LDHs and self-aggregating thick films reduce the catalytic ability of LDHs. Various strategies were developed to increase LDH conductivity and expand LDH nanostructures for enhanced DA detection.

One of the most important factors to improve in LDHs is electron mobility. Similar methods that improved the conductivity of LDH-based glucose sensors (see Section 4.1), such as metal doping and carbon compositing, can be applied to LDH-based DA sensors. As DA has very similar oxidation potentials to other biological molecules, including UA and AA, it is crucial to improve the electron mobility of LDHs for clearer distinctions between the similar biomolecules. On the other hand, if the LDH sensor conductivity is enhanced, the high catalytic ability of LDHs enables the simultaneous detection of DA and other biomolecules. Asif et al. synthesized a lamellar ZnNiAl-LDH/rGO composite for the simultaneous detection of DA, AA, and UA [42]. ZnNiAl–CO_3_-LDHs were synthesized via a hydrothermal method and converted into ZnNiAl–NO_3_-LDHs via ion exchange. The ZnNiAl-LDH was exfoliated in formamide and dispersed with exfoliated graphene oxide (GO) sheets for self-assembly into an LDH/GO superlattice. The LDH/GO composite was thermally reduced into LDH/rGO. The LDH/rGO material comprised flaky platelets with a 0.9 nm layer spacing and an increased basal spacing of 0.85 nm from 0.75 nm without the rGO. The addition of the rGO interlayers improved the conductivity, decreasing the charge transfer resistance from 1318 Ω to 745 Ω. The rGO also increased the surface area and porosity of the nanomaterial for greater catalytic ability. The ZnNiAl-LDH/rGO sensor’s detection limits were 0.1 nM for DA, 0.9 nM for UA, and 13.5 nM for AA. The LDH-based sensor exhibited excellent anti-interference from glucose and other common biomolecules within the 1–1000 nM linear detection range. The rGOs were crucial in providing a boost in conductivity and porous nanostructure for rapid ion diffusion. The electrooxidation peaks for DA, UA, and AA increased, resulting in clear peak separation in the CV curve. Same group grown CuMn LDH on CNT to detect H_2_S from live cell [126]. They have also developed a DA sensor with simultaneous biomolecule detection abilities by improving the electrocatalytic ability of ZnAl-LDHs with MWCNTs [127]. The sensor detected DA, bisphenol A, and acetaminophen while selecting against glucose and AA. A ZnAl-LDH with intercalated clopyralid anions was synthesized via co-precipitation under a nitrogen atmosphere. Multi-walled carbon nanotubes (MWCNTs) were mixed with the ZnAl-clopyralid-LDH. The resulting ZnAl-clopyralid-LDH/MWCNT composite comprised flaky ZnAl-LDHs, with MWCNTs integrated well with the LDH. The 5 wt% ZnAl-clopyralid-LDH/MWCNT sensor exhibited a higher peak electrooxidation current, owing to the excellent catalytic activity of the ZnAl-clopyralid-LDHs and high carbon content. Further increasing the LDH content increased the layer thickness, resulting in decreased current response due to decreased electron mobility. SWV found that the ZnAl–clopyralid-LDH/MWCNTs simultaneously detected DA, bisphenol A, and acetaminophen with LODs of 0.17, 0.18, and 0.14 μM, respectively. The linear detection ranges of the analytes were 7–500 μM for DA, 3–500 μM for bisphenol A, and 30–500 μM for acetaminophen. The composite LDH probe exhibited high selectivity against other organic compounds, including AA, sodium salicylate, glucose, sucrose, glutamic acid, and captopril, even with 25-fold more interfering species. The clopyralid intercalants likely shifted the electrooxidation potential window of the ZnAl-LDHs to encompass DA, bisphenol A, and acetaminophen but not the other biomolecules. Moreover, MWCNTs are endowed with excellent electron mobility, increasing the oxidation peaks of DA, bisphenol A, and acetaminophen such that the peaks were distinct under SWV analysis. The fact that a high MWCNT content had a greater impact on the peak oxidation potential than high ZnAl-LDH content emphasized the importance of the increasing conductivity for maximum DA detection sensitivity.

While compositing LDHs with carbon materials is a facile and effective method, other more unusual strategies have been used for improving LDH-based DA sensors. An interesting method homogeneously involves doping catalytic metal hydroxides. Zhang et al. doped NiCo-LDHs with Ni(OH)_2_ nanoboxes for improved DA detection [128]. Thin NiCo-LDHs were synthesized via a hydrothermal process and reacted with NaOH and CuCl_2_ to yield CuO nanocubes. Ni(OH)_2_ nanoboxes were formed from the CuO nanocubes via coordinate etching and precipitation, resulting in a Ni(OH)_2_/NiCo-LDH nanocomposite. The hybrid NiCo-LDHs comprised small Ni(OH)_2_ nanoboxes homogeneously dispersed on the thin NiCo-LDH flower petal-like nanosheets. The Ni(OH)_2_ nanoboxes possessed thin 30–40 nm walls enclosing a hollow interior. The NiCo-LDHs provided a porous nanostructure for abundant Ni(OH)_2_ adsorption. While the Ni(OH)_2_ nanoboxes themselves are not highly conductive, the homogeneously dispersed Ni(OH)_2_ nanoboxes possessed large high surface areas that were accessible for DA electrooxidation. The synergistic effects between the NiCo-LDH nanosheets and Ni(OH)_2_ nanoboxes improved electron mobility compared to bare NiCo-LDHs and bare Ni(OH)_2_ nanoboxes. The enhancements were indicated with chronoamperometry that determined a wide linear range between 0.05 and 1080 μM with a 17 nM LOD.

Another interesting method of improving the electron mobility in LDHs is phosphorization. Metal phosphides, such as Ni_2_P and Ni_x_Co_y_P, have exhibited excellent catalytic ability, owing to the P atom altering the electronic structure for more metallic character [129]. Thakur et al. phosphorized a NiFe-LDH for enhanced conductivity, improving the catalytic activity for electrochemical DA detection [130]. A phosphorized NiFe-LDH (NiFeP) was synthesized via a microwave-induced phosphorization reaction of NiFe-LDHs with red phosphorus. The NiFeP comprised thin, vertically aligned, and cross-linked nanosheets that aggregated into a 3D microflower structure. The NiFeP exhibited a lower charge transfer resistance than NiFe-LDHs, resulting in faster electron mobility for improved DA electrooxidation, indicated by the 2-fold-higher current response for the phosphorized NiFe-LDH than the regular NiFe-LDH. The SWV of the NiFeP DA probe determined a low LOD of 0.57 nM, with three distinct linear response ranges with varying sensitivities: 0.01–1 μM with a sensitivity of 427 μA mM^−1^ cm^−2^, 1–50 μM with a sensitivity of 32 μA mM^−1^ cm^−2^, and 100–500 μM with a sensitivity of 3.4 μA mM^−1^ cm^−2^. The NiFeP-LDH demonstrated high selectivity against AA, glucose, or UA, even at high concentrations of 1000, 3000, and 200 μM, respectively. The NiFeP-based DA sensor exhibited excellent stability, with no significant change to its morphology and electrochemical performance after 100 cycles. Ni-based LDHs have been modified to have unique morphologies and compositions for enhanced electrochemical DA detection.

LDH-based sensors have also been used to host spectroscopically active compounds for optical DA detection. Shi et al. intercalated a MgAl-LDH with N,N-Bis (carboxymethyl) aminomethylfluorescein (calcein) for CL DA detection [131]. The MgAl–calcein-LDH was synthesized via a solvothermal process and subsequently coated on ITO glass as a thin film. The LDH comprised horizontally or vertically grown nanosheets with a 30 nm thickness and 0.5 μm height, forming an interconnected, maze-like microstructure. The calcein was oxidized by the hydroxides in the LDHs and subsequently reduced by DA, emitting chemiluminescence. The MgAl–calcein-LDHs with a 1.25% calcein mole fraction exhibited a peak emission intensity at a wavelength of 510 nm. Further increasing calcein content reduced the CL intensity and red-shifted the emissions to 530 nm due to calcein aggregation. The MgAl–calcein-LDH exhibited a linear detection range of 0.5–101 μM with a detection limit of 0.352 μM. The calcein-based LDH also demonstrated high selectivity for DA, even when detecting DA in the presence of 500-times-more AA. While no CL response was measured, the vertically aligned MgAl–calcein-LDH nanowalls exhibited a two-times-higher response current than the horizontally stacked MgAl-calcein-LDHs according to CV analysis. The vertically aligned architecture improved electron and mass transport for enhanced DA electrooxidation. This result showed the advantage of more accurate electrochemical detection methods. More importantly, the result emphasized the necessity of optimizing LDH morphology for optimal electrochemical response but not necessarily for optical detection.

Many LDH-based materials were developed with excellent analytical performance for DA detection, owing to their high catalytic activity and versatile hosting abilities. The performance of many LDH-based DA sensors is summarized in Table 2. Improving the conductivity of LDHs with carbonaceous materials or doping other catalytic molecules yielded greater sensitivity for electrochemical DA detection. The stability of spectroscopically active molecules in the LDH also enables optical DA sensing.

### 4.3. H_2_O_2_

Monitoring H_2_O_2_ concentrations is valuable as H_2_O_2_ plays a critical role in many biological and industrial processes [132]. Current methods for H_2_O_2_ detection are prohibitive, requiring expensive precious metals and large analytical instruments [133]. Various LDHs have shown promise as low-cost electrochemical and optical H_2_O_2_ sensors, owing to their excellent catalytic and hosting abilities. Electrochemical H_2_O_2_ detection often relies on catalytic Co and Ni transition metals with conductive metal dopants or redox molecules to improve electron mobility. However, it is possible to employ reliable H_2_O_2_ detection with LDHs alone if the structure and conductivity of the LDHs are optimized.

High crystallinity of LDHs is crucial as purer LDH crystal phases with high crystallinity facilitate electron transfer during electrochemical redox. The ability to improve crystallinity via heat treatment and aging has already been thoroughly discussed (see Section 2.2 and Section 2.3). Here, controlling the purity of the crystal phases via tuning the metal ratios is discussed with an exemplary study. Farhat et al. optimized the ratio between Co and Mn in CoMn-LDHs synthesized via co-precipitation [40]. Co_3_Mn-LDH (Co-to-Mn ratio of 3:1) exhibited the purest LDH crystal phase. Increasing Co content resulted in cobalt hydrate crystal phases, whereas Mn-rich compositions yielded manganese carbonate phases. Most Co_x_Mn-LDHs close to the Co-to-Mn ratio of 3:1 comprised regular hexagonal plates with a 40–60 nm thickness and 200–580 nm lateral length. However, Co_1_Mn-LDHs (Co-to-Mn ratio of 1:1) and Co_5_Mn-LDHs (Co-to-Mn ratio of 5:1) resulted in irregularly sized nanosheets with significant aggregation, owing to impurities in their crystal phases. Thus, the presence of alternate crystal phases reduces the electron mobility and surface area, resulting in poor electrochemical redox and analyte adsorption, respectively. Co_3_Mn-LDHs exhibited the highest electrochemical response due to the purity of its LDH crystal phase. Based on chronoamperometric analysis, the optimized Co_3_Mn-LDH-modified probe possessed a linear range of 0.11–1.2 mM, an LOD of 86 μM, a sensitivity of 20 μA mM^−1^ cm^−2^, and selectivity against glucose, phosphate, and ascorbate. Controlling the crystal phases and crystallinity of the LDHs enables electrochemical H_2_O_2_ detection without dopants.

Because electrochemical LDH-based probes require LDHs to be applied to conductive electrodes, such as GCE, the increased impedance from electrode fabrication must be minimized. The main strategies of reducing impedance involved thinner LDH films and binder-less LDH adhesion. Briefly, in terms of LDH film thickness, thinner films allow the faster diffusion of H_2_O_2_ and other involved redox species in and out of the LDHs. However, a thick enough film was required for sufficient LDH-catalyzed H_2_O_2_ oxidation. The thickness of the LDH films is easily tuned by controlling the LDH loading. In the previously discussed study by Farhat et al., increasing the Co_3_Mn-LDHs loading from 10 to 20 μg increased film thickness and electrochemical response [40]. Doubling the LDH loading to 40 μg decreased the electrochemical response current and decreased electrode stability.

**Figure 7:**
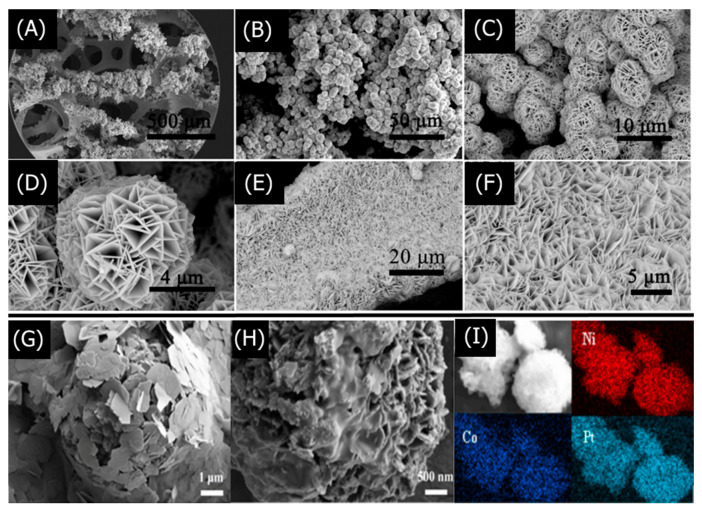
SEM micrographs of the outer surfaces of the NiFe-LDH/Ni foam at (**A**,**B**) low and (**C**,**D**) high magnifications, and the inner surfaces at (**E**) low and (**F**) high magnifications, reproduced from reference [134] with permission from Elsevier B.V., 2018. SEM micrographs of (**G**) bare NiCo-LDH and (**H**) Pt-doped NiCo-LDH; (**I**) elemental mapping of Ni, Co, and Pt in the Pt-doped LDH nanocomposite, reproduced from reference [52] with permission from Elsevier, 2021.

The LDH-electrode impedance can also be decreased via binder-less adhesion of catalytic LDHs on conductive substrates for dopant-less H_2_O_2_ detection. You et al. grew NiFe-LDHs on Ni foam via in situ hydrothermal urea hydrolysis [134]. The NiFe-LDH/Ni foam exhibited a bronze color due to the nucleation of spherical NiFe-LDH microspheres on the outer foam surface. SEM micrographs in Figure 7 revealed the formation of different LDH nanostructures, depending on the location on the Ni foam. On the outer portion of the Ni foam, the NiFe-LDH/Ni foam electrode comprised cross-linked nanosheets that produced a porous, flower-like structure (Figure 7A–D). However, the inner surfaces of the Ni foam comprised vertically aligned NiFe-LDH nanosheets instead of nanospheres (Figure 7E,F), suggesting different growth mechanisms depending on the diffusion rate of the metal ions. Electron transfer was enhanced because the NiFe-LDHs were directly synthesized on the Ni foam, resulting in a high sensitivity of 1704 μA mM^−1^ cm^−2^. Chronoamperometry determined a linear detection range of 0.5–840 μM with a low detection limit of 0.5 μM. Moreover, the structure of the conductive substrate can be utilized to enhance catalytic ability. The porous architecture of the Ni foam enabled the fast diffusion of O_2_, a byproduct of H_2_O_2_ oxidation. The sensor demonstrated excellent selectivity against DA, UA, and glucose. The in situ growth of LDH nanosheets on a conductive substrate improves electron transfer and ion diffusion for enhanced H_2_O_2_ detection.

While electrochemical methods are sensitive and accurate, optical methods may be more useful for naked-eye applications. Thus, colorimetric reactions, such as the TMB-H_2_O_2_ system, are common for fast and equipment-less H_2_O_2_ detection. TMB is a chromogenic compound that turns blue when oxidized by H_2_O_2_ when exposed to peroxidase-like LDHs. The colorimetric response may be enhanced by increasing the catalytic ability of LDHs. One method of improving the optical response is by exfoliating LDHs for many active sites and abundant TMB adsorption. Zhan et al. exfoliated thin NiFe-LDH nanosheets with TMB intercalants for the colorimetric detection of H_2_O_2_ [50]. Bulk NiFe–CO_3_-LDHs underwent ion exchange to yield NiFe–NO_3_-LDHs that were more easily exfoliated in L-asparagine, owing to weaker intermolecular forces between the brucite-like layers. The exfoliated LDHs comprised 2 nm-thick and irregularly sized nanoflakes instead of the typical regular hexagon shape of bulk LDH. A higher H_2_O_2_ concentration yielded a darker blue solution. The exfoliated NiFe-LDH exhibited an absorbance almost 4-fold-higher than that of bulk NiFe-LDH, owing to increased peroxidase-like activity. The NiFe-LDH-based H_2_O_2_ sensor exhibited a linear determination range of 0.01–0.5 mM with an LOD of 4.4 μM. The exfoliation of bulk LDHs yield smaller nanoparticles with a higher surface area and more active sites for enhanced catalytic activity.

LDHs can also be doped with catalytic materials that may also improve the structure of LDHs for increased optical response to H_2_O_2_. Cao et al. synthesized Pt-doped CoNi-LDHs for colorimetric H_2_O_2_ detection [52]. The LDHs were synthesized directly onto Ni foam via a hydrothermal process and immersed in a Na_2_PtCl_6_ solution for Pt doping. The pristine CoNi-LDHs comprised a flaky nanostructure (Figure 7G). In contrast, the Pt-doped CoNi-LDHs comprised a more densely cross-layered porous structure (Figure 7H) with Pt nanoparticles homogeneously adsorbed by the LDH nanosheets (Figure 7I). The Pt facilitated TMB electrooxidation when exposed to H_2_O_2_, whereas the CoNi-LDH alone could not. Interestingly, the highly catalytic CoNi-LDHs exhibited poor peroxidase-like ability contrary to a previous study [62]. Thus, catalytic dopants may be essential for consistent optical H_2_O_2_ detection. The colorimetric probe exhibited a linear detection range of 10–90 mM and a detection limit of 0.76 mM. An additional benefit of the TMB-H_2_O_2_ system is the secondary ability to detect glutathione, as glutathione reverts the oxidized blue TMB to colorless TMB. The Pt-doped CoNi-LDH detected glutathione concentrations in a linear detection range of 50–500 mM. Colorimetric H_2_O_2_ sensing using LDHs is fast and requires no additional equipment for simple detection purposes. The optical response may be enhanced by exfoliating LDHs for a higher surface area and adding catalytic dopants for more reliable TMB oxidation.

Ni and Co-based LDHs are excellent catalysts for sensitive electrochemical H_2_O_2_ detection. Their catalytic abilities may be optimized by improving crystallinity and directly synthesizing the LDHs onto conductive substrates. Furthermore, LDHs can host or directly catalyze spectroscopically active molecules for optical H_2_O_2_ detection. The performance of recent LDH-based H_2_O_2_ sensors is summarized in Table 3.

### 4.4. Nitrogen-Based Toxins

Various nitrogen-based toxins, such as ammonia, nitrogen oxides, nitrites, and melamine, are prolific due to industrialization and urbanization. Thus, the detection of these nitrogen-based toxins is essential for protecting the environment and preventing severe illnesses. LDHs are versatile materials that can effectively capture nitrogen-based toxins in the air or water for electrochemical or optical detection.

Nitrogen-based gasses, such as ammonia and nitrogen oxides, are dangerous even at low concentrations. For gas sensors, high porosity is one of the most important characteristics as it provides many active sites for electrochemical detection. Ammonia is a nitrogen-based, toxic gas that is frequently used in industrial and agricultural sectors. However, exposure to over 25 ppm of ammonia can damage people’s eyes, skin, liver, and respiratory tract [135]. LDHs are promising host materials, owing to their high adsorption capacity, high surface area, and ability to host ammonia-reacting molecules, such as PANI. Qin et al. prepared a porous ZnTi-LDH/PANI composite for ammonia detection via a hydrothermal process [136]. ZnTi-LDH possessed a porous 3D framework due to the partial decomposition of the hierarchical ZnTi-LDH nanosheets during the acidic polymerization of the aniline monomers. Exposure to ammonia increased the sensor’s resistance because the ammonia deprotonated PANI, resulting in a conversion from a conductive emeraldine salt form to a less conductive emeraldine base form. The ZnTi-LDH provided a porous structure that hosted abundant PANI for sensitive detection and allowed fast ammonia diffusion, resulting in high sensitivity with a low LOD of 200 ppb. Increasing ammonia exposure resulted in higher probe resistance, which could be calibrated for two linear ranges: 0.2–2 ppm and 2–50 ppm. The sensor also demonstrated high selectivity against interfering gasses, such as methane, hydrogen, methanol, and acetone. Due to the stable 3D architecture of the ZnTi-LDH, the composite probe exhibited high stability, indicated by the 88.4% response retention after 15 days.

Nitrogen oxides, such as NO_2_, are toxic gasses that threaten humans and the environment with health complications, acid rain, and photochemical smog. LDHs are promising gas sensors for NO_2_, owing to their excellent stability and catalytic activity towards NO_2_ [137]. While LDHs alone provide a large surface area for gas detection, LDHs may be composed of highly porous carbon nanostructures for improved conductivity and increased surface area. Qin et al. synthesized a ZnTi-LDH/rGO composite to detect NO_2_ gas [138]. A ZnTi-LDH was first synthesized via a solvothermal reaction involving TiCl_4_, Zn(NO_3_)_2_·6H_2_O, and GO precursors in a urea/ethanol aqueous solution. The resulting composite comprised flower-like LDH nanosheet stacks grown on larger rGO nanosheets. LDH-rich compositions yielded thicker LDH nanosheet stacks, so the addition of sufficient rGO was critical in growing homogeneously spaced LDH crystal nanosheets. The highly porous structure and large surface area (275 m^2^ g^−1^) afforded by the rGO and LDH nanosheets enabled effective gas sensing, enabling unhindered diffusion and abundant surface active sites. The rGO also enhanced the probe’s sensitivity response by improving the conductivity of the LDH composite. Exposure to NO_2_ gas decreased the resistance of the ZnTi-LDH/rGO probe, exhibiting a 0.2–10 ppm linear response range, a 50 ppb detection limit, a <2 s response time, and a 189 s recovery time. Conclusively, LDHs that are excellent host materials can be further enhanced for electrochemical gas sensing by combining with conductive and porous carbon nanostructures.

Melamine is a nitrogen-based compound commonly used in plastics, adhesives, and coatings, but overexposure to melamine can cause dangerous and even fatal damage to the renal system [139]. While LDHs may not directly react with melamine, LDHs are excellent hosts for fluorescent compounds, such as dye-functionalized Ag nanoparticles. The primary issue that LDHs alleviate is the self-aggregating property of Ag nanoparticles that reduce fluorescence. Ren et al. synthesized a composite bilayer thin-films comprising MgAl-LDHs and Ag/chromotropic acid (CTA) nanoparticles for melamine detection [51]. Exfoliated MgAl-LDHs and Ag–CTA nanoparticles were deposited onto a quartz glass substrate via alternating dip-coating. The resulting thin film comprised 2 nm-thick bilayers of MgAl-LDH/Ag–CTA nanoplatelets. The LDHs increased the fluorescence response by almost 2-fold because the LDHs immobilized the Ag–CTA to reduce agglomerations for more reactive sites and reduced non-radiative states. The fluorescent intensity linearly increased with increased melamine concentrations in a linear range of 30–100 nM, with a low LOD of 4 nM. Standard addition assays of melamine in milk found high recoveries between 97.5% and 102.3% with an RSD < 2.45%. LDHs are excellent hosts for spectroscopically active compounds that tend to aggregate.

**Figure 8:**
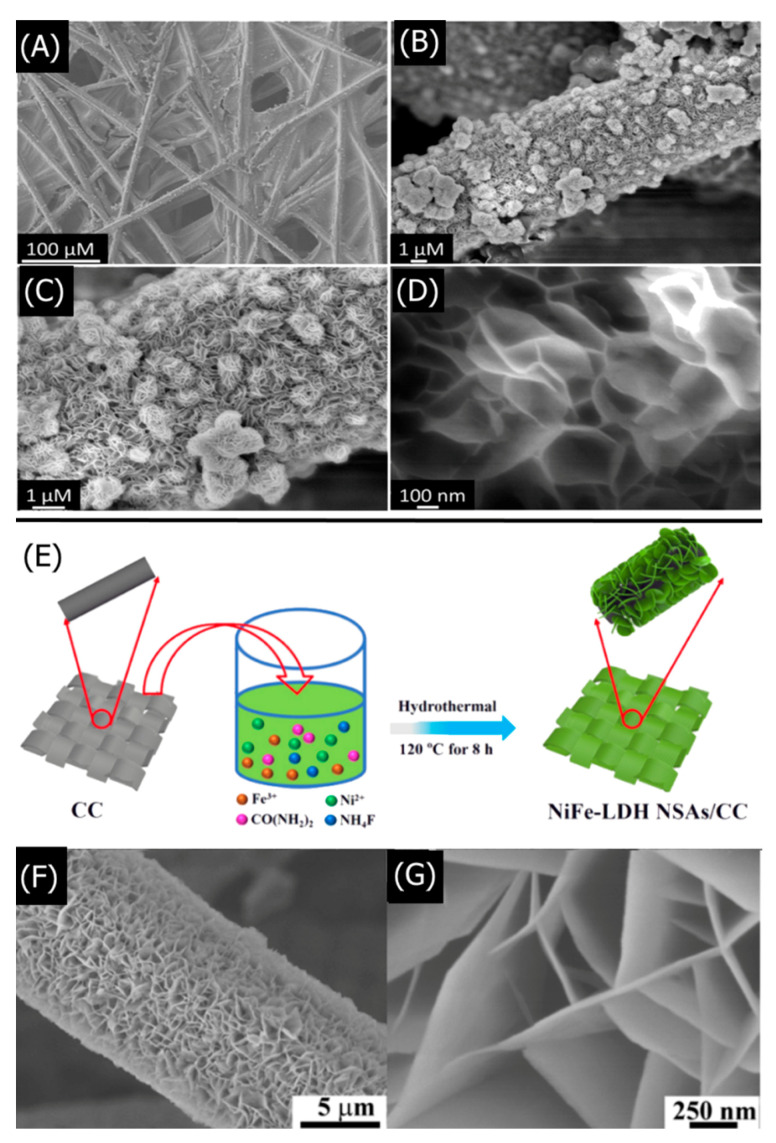
(**A**–**D**) SEM micrographs of MgAl-LDH/carbon paper at different magnifications, reproduced from reference [141] with permission from Elsevier B.V., 2019; (**E**) schematic illustration of the synthesis of the NiFe-LDH/carbon cloth composite; (**F**) low- and (**G**) high-magnification SEM micrographs of the NiFe-LDH/carbon cloth composite, reproduced form reference [142] with permission from American Chemical Society, 2018.

Nitrites are nitrogen-based ions commonly used as food additives or corrosion inhibitors. However, overexposure to nitrites can cause hemoglobin damage and cancer [140]. Thus, the affordable and accurate detection of nitrites in water is essential. Nitrites may be electrochemically detected via an electrochemical redox reaction with various LDHs. These LDHs, however, are often composited with carbon nanostructures for increased conductivity and surface active sites. Xiang et al. developed a MgAl-LDH-based sensor for electrochemical nitrite detection [141]. MgAl-LDHs were grown in situ on carbon cloth via hydrothermal process, yielding 10 μm-diameter carbon nanofibers coated with flower-like MgAl-LDH nanosheets (Figure 8A–D). The LDH nanoflower growths (Figure 8C) afforded abundant active sites for ion adsorption, and the porous 3D architecture enabled fast ion diffusion. Based on CV analysis, the sensor exhibited a linear detection range of 3.7–117.4 μM and a low 30 nM detection limit. The LDH-based probe demonstrated high selectivity against common interfering anions such as SO_4_^2−^, CO_3_^2−^, and Cl^−^, even at 100-fold the nitrite concentration. In a similar work, Ma et al. fabricated a NiFe-LDH/carbon cloth composite probe for electrochemical nitrite detection [142]. The vertically aligned NiFe-LDH nanosheets were synthesized via an in situ hydrothermal method with NH_4_F (Figure 8E). The LDHs comprised 15–20 nm-long nanosheets perpendicular to the carbon nanofiber base (Figure 8F,G).

Decreasing the Ni-to-Fe ratio resulted in thinner LDH nanosheets and decreased surface areas from 7.2 cm^2^ (Ni-to-Fe ratio of 5:1) to 5 cm^2^ (Ni-to-Fe ratio of 1:3). NiFe-LDH with a Ni-to-Fe ratio of 3:1 produced the maximum nitrite oxidation current owing to its high electroactive surface area and Ni content. Chronoamperometric analysis determined a 5–1000 μM linear detection range, a sensitivity of 803.6 μA mM^−1^ cm^−2^, 3 s response time, and a 20 nM detection limit. In both studies, the carbon nanofiber template reduced LDH aggregation by providing well-spaced nucleation sites. The porous 3D fiber structure enabled fast ion diffusion and abundant active sites for faster redox kinetics. Moreover, binder-less probe fabrication reduced electrical impedance, increasing electron mobility for a higher peak response current. While the carbon nanofiber structure was the template for LDH growth, the nanostructure of the LDHs was optimized for a high surface area by manipulating the metal ratios.

LDH materials are promising sensor materials for different nitrogen-based gasses because of their high adsorptive capability and catalytic activity. LDHs are excellent hosts that can adsorb various spectroscopically active compounds, enabling the optical detection of nitrogen-based toxins. Electrochemical LDH-based sensors have excellent catalytic reactivity with some nitrogen-based toxins. Their catalytic ability can be improved by directly synthesizing the LDHs onto porous and conductive carbon substrates and tuning the metal ratios in the LDHs for maximum surface area. The different strategies for improving LDH-based electrochemical and optical detection for nitrogen-based toxins are summarized in Table 4.

### 4.5. Metal Ions

Hg^2+^ and other metal ions are toxic pollutants that can cause severe environmental and biological harm—even at low concentrations. Thus, various LDH sensors have been designed to detect low concentrations of various metal ions using both electrochemical and optical sensing methods. Because the presence of these metal ions in drinking water is especially hazardous for humans, both detection and extraction are desired. LDHs enable the simultaneous extraction and detection of heavy metal ions with their highly lamellar structure and excellent hosting ability. Shamsayei et al. synthesized ZnCr-LDHs intercalated with NO_3_^−^ and Nafion for Hg^2+^ extraction and detection [143]. The resulting LDHs comprised irregularly sized nanoplates with a large basal spacing of 24.65 Å, owing to the Nafion intercalants, as well as an LDF surface from 49.92 to 61.50 m^2^ g^−1^. The ZnCr–Nafion-LDHs exhibited a high Hg^2+^ adsorption capacity of 302.14 mg g^−1^. The high surface area exposed many active hydroxyl sites for Hg^2+^ adsorption. The sulfonate groups in the Nafion exhibited a high adsorption affinity for Hg^2+^. Its extraction ability was calibrated for Hg^2+^ detection. The ZnCr–Nafion-LDH sensor exhibited a linear detection range of 0.013–500 μg L^−1^ and a low detection limit of 4 ng L^−1^. The sensor demonstrated high reproducibility with a low RSD under 4.2% for five repeated measurements in one day. The ZnCr–Nafion-LDH also exemplified good stability, as indicated by the 5.8% RSD for five daily measurements. The extraction-based detection mechanism should be considered if the sensors are expected to be deployed for high-risk applications such as ensuring the safety of human drinking water.

LDH-based optical sensors are also advantageous for high-risk applications, owing to their visual indication of heavy metal ions. The excellent hosting abilities of LDHs are promising for improving fluorescent emissions. Chen et al. synthesized a Mg_2_Al-LDH/primuline dye composite thin-film probe for fluorescent Hg^2+^ detection [144]. Mg_2_Al-LDHs synthesized via a hydrothermal process were exfoliated in formamide. An alternating dip-coating method deposited bilayers of Mg_2_Al-LDH and primuline dye on a clean quartz glass substrate. The resulting thin-film probe comprised a uniform and smooth surface with 2.48 nm-thick LDH-primuline bilayers. The LDH-primuline exhibited enhanced fluorescence intensity compared to bare primuline, owing to the LDHs suppressing the non-radiative rotation and vibration energy states of the primuline. The LDH layer also reduced primuline aggregations for increased fluorescence intensity. The Mg_2_Al-LDHs also shielded the primuline dye from UV-induced bleaching, increasing the decay time from 50 min to 10 h. The LDH with 25 bilayers exhibited an excellent 0.13 pM LOD, a wide linear detection range of 2.5–100 nM, and a fast response rate. Increasing the film thickness reduced the fluorescent response due to the slower Hg^2+^ diffusion. LDHs are highly adsorptive nanomaterials that enable effective Hg^2+^ detection by hosting spectroscopically active guest dyes.

**Figure 9:**
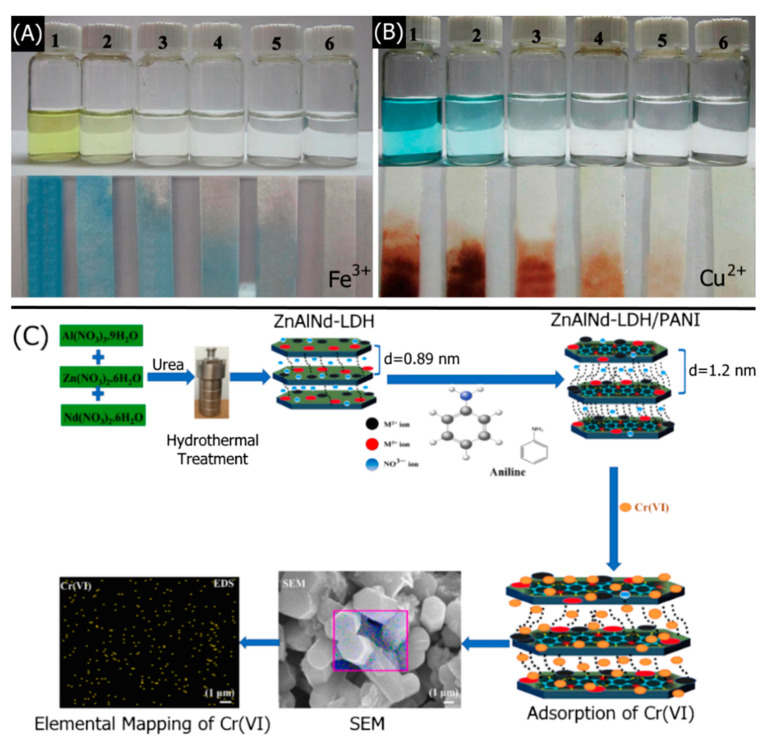
Color change of filter paper with MgAl–Fe(CN)_6_-LDHs when submerged in an (**A**) Fe^3+^ and (**B**) Cu^2+^ solution at different concentrations: 0.1 M, 0.01 M, 1 mM, 0.1 mM, 0.01 mM, and 1 nM for vials 1–6, respectively, reproduced from reference [145]; with permission from Elsevier B.V., 2016 (**C**) schematic illustrating the synthesis of the ZnAlNd-LDH/PANI nanocomposite and its adsorption of Cr^6+^. The SEM micrograph of the LDH/PANI and the elemental mapping of the adsorbed Cr^6+^ are also shown, reproduced from reference [146] with permission from Elsevier B.V., 2021.

LDH-based optical sensors were also fabricated to detect multiple metal ions that pose serious health risks. Wang et al. fabricated an MgAl-LDH-based colorimetric sensor that detected Fe^3+^, Cd^2+^, Cu^2+^, and Pb^2+^ [145]. The MgAl–NO_3_-LDHs were directly synthesized onto the filter paper via a hydrothermal method and intercalated Fe(CN)_6_^4−^ or S^2−^ via ion exchange. The resulting MgAl–anion-LDH comprised cellulose fibers from the filter paper coated with disc-like nanosheets approximately 20 nm thick and 100–200 nm in diameter. Depending on the intercalated anion, the concentrations of Fe^3+^, Cd^2+^, Cu^2+^, and Pb^2+^ could be determined via colorimetric analysis. MgAl–Fe(CN)_6_-LDHs were initially yellow but turned blue when exposed to Fe^3+^ (Figure 9A) and brown when exposed to Cu^2+^ (Figure 9B). Similarly, the MgAl–S-LDHs were initially clear but turned grey when exposed to Pb^2+^ and yellow when exposed to Cd^2+^. Increased metal ion concentrations produced a darker color, and having multiple metal ions present resulted in one dominant color, suggesting an imbalance of attraction between the intercalated anion and each analyte. LDHs with either Fe(CN)_6_^4−^ or S^2−^ demonstrated high stability, producing the same color-depth after eight months of exposure to air. Because the colorimetric MgAl-LDH sensor exhibited poor selectivity and no precise way to determine the metal ion concentrations, this detection method is not advised for high-accuracy applications. Rather, this type of multi-analyte sensor is useful for detecting poisonous metal ions in drinking water or other applications that require an immediate and obvious indication of many harmful substances.

Indeed, one of the most valuable properties of LDHs is their ability to host detection-enabling molecules. While their ability to host fluorescent or color-inducing anions for metal detection has been discussed, their ability to host multi-purpose molecules must be emphasized. Dyes are frequently doped into LDHs for optical detection, but the redox reactions of various dyes produce electrochemical signals. Lajevardi Esfahani et al. fabricated a simultaneous electrochemical and optical Al^3+^ sensor via a layer-by-layer assembly of MgAl-LDHs and alizarin red S (ARS) on an ITO/PET substrate [57]. A five-layer electrode exhibited the lowest sheet resistance, with successive layers decreasing the conductivity and ion diffusion. The Al^3+^ reacted with the ARS to yield an ARS–Al^3+^ complex, which was detected as a distinct oxidation peak. The MgAl-LDH-based electrochemical sensor exhibited a linear response range of 0.2–120 μM, a low detection limit of 10.1 nM, and excellent selectivity against other metal ions such as Co^2+^, Ni^2+^, and Cu^3+^. The adsorbed ARS molecules simultaneously functioned as the fluorescent probe molecule. The immobilized ARS molecules exhibited increased fluorescence intensity when immobilized by the LDH compared to when in solution, owing to the LDH suppressing nonradiative states. Increasing Al^3+^ exposure increased the fluorescence intensity, resulting in the same linear detection range of 0.2–120 μM with a 23 nM detection limit, significantly higher than the electrochemical LOD. Many optical detection strategies have been developed, but electrochemical methods may allow for more sensitive detection with lower LODs. Sensor studies involving LDHs–dye interactions for optical metal ion detection should also test for an electrochemical response.

Some LDHs also possess an innate fluorescence response that can be used for optical metal ion detection. LDHs often host fluorescent molecules or catalyze fluorescent reactions that emit light when exposed to the analyte. However, the natural fluorescence of some LDHs can be used in reverse, wherein the analyte absorbs some irradiation to reduce the fluorescent reaction from the LDHs. Wani et al. synthesized a trimetallic ZnAlNd-LDH supported by a PANI template for the fluorometric Cr^6+^ detection via a hydrothermal urea hydrolysis method—as illustrated in Figure 9C [146]. The trimetallic ZnAlNd-LDH/PANI nanocomposite exhibited a more porous structure, owing to fewer agglomerations because of Nd^3+^ integration. The acid polymerization of aniline into PANI reduced LDH aggregation while simultaneously forming long PANI nanorods integrated into the LDH via strong electrostatic interactions. The ZnAlNd-LDHs exhibited a low fluorescence intensity, owing to their smooth surface topology. In contrast, the ZnAlNd-LDH/PANI exhibited a seven-fold-higher fluorescence intensity than the ZnAlNd-LDH due to the PANI forming abundant surface defects for photogenerated electron–hole recombination. Because Cr^6+^ absorbed light in the same frequency range as the ZnAlNd-LDH/PANI, an increase in Cr^6+^ decreased the fluorescence intensity. The fluorometric probe exhibited a 200–1000 ppb linear detection range with an 8 ppb detection limit. This absorption-type of fluorimetric detection may help selectively detect analytes with similar fluorescent emissions but different absorption ranges. The initial fluorimetric response can also be increased to yield a broader detection range.

The performance of different LDH-based sensors for different metal ions is summarized in Table 5. Various LDH nanomaterials were designed as effective catalysts and hosts for optical metal ion detection. In particular, LDH–dye guest–host interactions are often used for optical detection but may exhibit detectable electrochemical responses to metal ions. The high adsorptive property of LDH materials enables simultaneous metal ion extraction and detection for high-risk applications. Some LDHs exhibit natural fluorescence, which can be used for detecting compounds with similar absorption spectra.

### 4.6. Organic Compounds

The detection and monitoring of organic molecules, such as naphthol, vitamin B6, and ethanol, are critical as these compounds play significant roles in the environment and human health. LDHs are easily adaptable nanomaterials that can be modified to electrochemically or optically detect a wide range of organic molecules. While the metals in the LDHs used to detect the various organic compounds may differ depending on the analyte, the principles behind optimizing the electrochemical or optical sensing performance are generally applicable. Thus, this section focuses on methods of altering LDHs for the improved detection of organic molecules with exemplary organic molecules.

One of the largest drawbacks of implementing LDHs for detecting organic compounds is their poor conductivity and tendency to aggregate. These properties often prevent sensitive electrochemical detection at low concentrations. Exfoliating LDHs reduces agglomerations and improves their compatibility with other materials for improved performance. Wang et al. found that highly porous ZIF-67 MOFs could only be synthesized from exfoliated CoAl-LDHs used for the simultaneous detection of α- and β-naphthol [49]. CoAl–NO_3_-LDHs were synthesized via co-precipitation, exfoliated in formamide, and reacted with 2-methylimidazole, yielding exfoliated CoAl-LDH/ZIF-67 nanocomposites. Bulk CoAl-LDH/ZIF-67 comprised regular hexagon-shaped nanoparticles, typical of hydrotalcite-like compounds, with negligible ZIF-67 nucleation. In comparison, exfoliated CoAl-LDH/ZIF-67 exhibited a dodecahedral morphology, owing to abundant ZIF-67 growth. The ZIF-67 modification increased the effective area to 0.314 cm^2^ and lowered the charge transfer resistance to 70 Ω. Consequently, the exfoliated CoAl-LDH/ZIF-67 exhibited a higher peak oxidation current than bare LDH, ZIF-67, or bulk CoAl-LDH/ZIF-67. The exfoliated CoAl-LDHs possessed more accessible Co sites for integration with the MOF structure. Because minor variations of organic molecules—such as α- and β-naphthol—possess similar oxidation potentials, the improved electrocatalytic properties are necessary to yield distinguishable response peaks. DPV found that the exfoliated LDH/ZIF-67 sensor exhibited a low 62 nM LOD for α-naphthol with two linear detection ranges: 0.3–50 and 50–150 μM. The LOD for β-naphthol was higher at 94 nM with two linear detection ranges: 0.3–40 and 40–150 μM. The exfoliated LDHs can be more easily composited with other nanoparticles, altering their morphology and conductivity for enhanced analytical performance.

The improved ability to compose exfoliated LDHs is further exemplified by Zhan et al., who composed exfoliated Co_2_Al-LDH with rGO to immobilize hemoglobin for electrochemical trichloroacetic acid detection [147]. The Co_2_Al–CO_3_-LDHs synthesized via urea hydrolysis underwent ion exchange with NaNO_3_, followed by exfoliation in formamide. The ion exchange step was necessary because urea hydrolysis results in adhesive CO_3_^2−^ ions which prevent exfoliation. GO nanosheets were added to the exfoliated LDHs and reduced into rGOs with hydrazine monohydrate, yielding loosely held LDHs adsorbed onto the larger rGO nanosheets. Because the LDHs assembled onto the rGOs via intermolecular forces between the LDH and rGO sheets, LDH exfoliation increased the surface area for homogeneous adhesion and reduced self-aggregation. The rGOs were particularly important in improving the chemical stability of the exfoliated LDHs. A mixture of hemoglobin and LDH/rGO was deposited onto a CILE, yielding a hemoglobin-based biosensor. Hemoglobin was effectively immobilized in the LDH/rGO matrix via strong electrostatic interactions with the positively charged LDH and π–π stacking with rGO. The hemoglobin retained its biomolecular structure, indicating excellent biocompatibility with the LDH/rGO composite. The rGO improved electron mobility, and the LDH provided a large surface area for enhanced ion adsorption, resulting in a significantly lower electron transfer resistance and probe sensitivity than a bare hemoglobin/CILE electrode. The LDH/rGO composite provided a stable and conductive matrix for hemoglobin immobilization while the intercalated hemoglobin catalyzed the electroreduction of trichloroacetic acid. SWV was determined with a 0.82 mM LOD and a 2.5–410 mM linear detection range. The LDH/rGO provided a stable matrix which reduced hemoglobin denaturing, as indicated by the 92% response current retention after 4 weeks. LDHs alone would not have been a sufficiently conductive host for hemoglobin, resulting in significant adsorptive denaturing. Moreover, bulk LDHs would have yielded a denser lamellar structure with lower conductivity, which decreases biocompatibility. Thus, LDH exfoliation is useful when composing LDHs of other materials to increase their surface area and conductivity for more sensitive electrochemical detection.

While exfoliation is beneficial in compositing with carbon nanostructures, bulk LDHs still synergize well with various carbon materials. If a simpler synthesis process without any complex intercalation is desirable, then bulk LDHs may be comprised of carbon materials for improved conductivity, resulting in enhanced electrochemical detection. Wang et al. doped NiAl-LDHs with carbon quantum dots (CQDs) as an effective sensor for acetylcholine [148]. The NiAl-LDHs synthesized via a hydrothermal process were mixed with varying amounts of CQDs. The resulting nanocomposite comprised cross-linked NiAl-LDH nanosheets that formed flower-like microspheres. The porous LDH nanosheets adsorbed the CQDs, owing to the strong electrostatic interactions between the negative surface charge of the CQDs and the positive surface charge of the brucite-like LDH layers. Increasing the CQD content to a CQD:LDH ratio of 0.025 enhanced the peak oxidation current, owing to increased conductivity. Moreover, the oxygen-containing functional groups on the CQDs attracted the positively charged acetylcholine, increasing acetylcholine adsorption onto the LDH nanosheets. The higher CQD content decreased the response current due to increased water solubility and lower NiAl-LDHs. Chronoamperometric analysis of the LDH/CQD probe determined a linear response range of 5–6885 μM, a sensitivity of 133.2 μA mM^−1^ cm^−2^, and an LOD of 1.7 μM. The CQDs-doped NiAl-LDH demonstrated good selectivity against negatively charged neurotransmitters with less than 12% current interference for DA, AA, and norepinephrine, unlike the CQD-less NiAl-LDH sensor, which suffered from interference as high as 61%. Compositing LDHs with small, negatively charged particles such as CQDs is an easy and environmentally friendly modification that enhances the selectivity and conductivity of LDH-based sensors.

**Figure 10:**
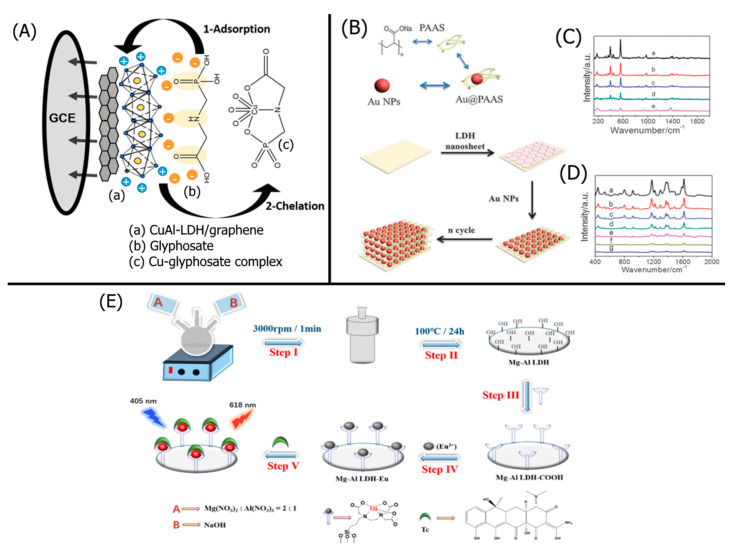
(**A**) Illustration of Cu oxidation suppression due to glyphosate adsorption followed by chelation, reproduced from reference [149] with permission from; MDPI, 2020; (**B**) schematic representation of the assembly of Au/PAAS/MgAl-LDH via a layer-by-layer assembly method. SERS detection toward (**C**) thiram ((**a**) 10^−3^ M, (**b**) 10^−4^ M, (**c**) 10^−5^ M, (**d**) 10^−6^ M and (**e**) 10^−7^ M), and (**D**) malachite green ((**a**) 10^−6^ M, (**b**) 10^−7^ M, (**c**) 10^−8^ M, (**d**) 10^−9^ M, (**e**) 10^−10^ M, (**f**) 10^−11^ M and (**g**) 10^−12^ M), reproduced from reference [150]. with permission from The Royal Society of Chemistry, 2015; (**E**) Schematic diagram of the synthesis and structure of the MgAl-LDH/Eu composite used for the colorimetric detection of tetracycline. Step I: Co-precipitation reaction among Mg(NO_3_)_2_·6H_2_O, Al(NO_3_)_3_·9H_2_O, and NaOH; Step II: Hydrothermal treatment for 24 h and MgAl-LDH was afforded; Step III: Silanization reaction with N-(trimethoxysilylpropyl) ethylenediamine triacetic (EDTA) acid sodium; Step IV: Encapsulation of europium ions and MgAl-LDH/Eu was obtained; and Step V: An “off−on” recognition process in the presence of tetracycline, reproduced from reference [53] with permission from American Chemical Society, 2019.

Another example of bulk LDHs compositing with conductive carbon structures involves integrating smaller LDHs into a porous graphene structure. Zhang et al. doped CuAl-LDH into a graphene template, resulting in increased conductivity and reactivity [149]. CuAl-LDHs synthesized via co-precipitation were dispersed with graphene nanosheets and drop-cast onto a GCE, yielding a glyphosate-sensing probe. The LDH/graphene nanocomposite comprised small CuAl-LDHs homogeneously dispersed on the 3D graphene structure. The LDH/graphene-modified electrode exhibited a low 54.5 Ω charge transfer resistance, lower than bare GCE (89.27 Ω). Furthermore, a pure LDH crystal phase was synthesized by tuning the Cu:Al ratio of 72:28, improving the nanocomposite’s conductivity. The CuAl-LDH/graphene nanocomposite exhibited the highest redox response, owing to the graphene’s increased conductivity and surface area. Exposure to glyphosate inhibited Cu oxidation during CV, indicated by the decrease in the oxidation peak. The glyphosate nearly doubled the charge transfer and adsorption resistances, owing to glyphosate chelation with the CuAl-LDH (Figure 10A). DPV found that the CuAl-LDH/graphene sensor exhibited a linear detection range of 2.96–1180 nM with a 1 nM detection limit. Standard addition assays with filtered water found high recoveries of 97.65–108.08%, highlighting the CuAl-LDH/graphene sensor’s promising detection ability. Bulk LDHs synergize well with large or small carbon nanoparticles and can be easily composited for enhanced analytical performance.

Porous carbon structures, such as graphene, used as templates for LDH crystallization, may be replaced with other highly porous structures. Yadav et al. synthesized a porous NiFe-LDH based on an Fe-MIL-88 MOF template for kojic acid detection [151]. A mixture of Fe-MIL-88 was reacted with Ni(CH_3_COO)_2_ in dimethylformamide under solvothermal conditions to yield NiFe-LDHs. The resulting MOF-based synthesis process yielded spindle-shaped nanoparticles that comprised a layered structure grown from the spindle-like MOF nanostructure. Electrochemical characterization using [Fe(CN)_6_]^3−/4−^ redox probe ions found a higher oxidation response current for the MOF-template-formed NiFe-LDHs than bare Fe-MIL-88. The high surface area made many active sites available and increased the adsorption capacity, resulting in a stronger electrochemical signal. Chronoamperometric analysis determined an LOD of 0.73 μM with a sensitivity of 32 μA mM^−1^ cm^−2^ and two linear ranges: 0.001–1.5 mM and 1.5–4.5 mM. The LDH-based probe demonstrated excellent stability, indicated by the low 5.6% RSD after three repeated measurements and 95% signal retention after 1 week at room temperature. The NiFe-LDH exhibited high selectivity, with most interferants causing less than 5% change to the response current. The standard addition of kojic acid to tomato sauces from three different companies found reliable recoveries between 86% and 106%. The morphology of the LDH plays a critical role in determining the analytical performance of an LDH-based electrochemical sensor. Thus, controlling the structure of LDH nanoparticles via template-based synthesis is an effective way of enhancing electrochemical detection.

For detecting hydrophobic analytes, LDHs can be exchanged with large organic anions for improved analyte adsorption and selectivity. Kameni et al. intercalated bis(ethylhexyl) hydrogen phosphate (BEHP) into NiAl-LDH layers for the selective detection of methyl parathion (MP) against the structurally similar 4-nitrophenol (4-NP) [152]. The NiAl–NO_3_-LDHs underwent ion exchange with BEHP, reducing the crystallinity of LDHs while increasing the interlayer spacing. Intercalation durations under 16 h resulted in the partial BEHP intercalation, whereas long intercalation times resulted in BEHP de-intercalation, owing to the poor BEHP solubility. For intercalating large and complex organic molecules into LDHs, the intercalation duration must be optimized for maximum anion adsorption. Optimizing the ion exchange duration is especially critical for intercalating hydrophobic molecules such as BEHP. The NiAl–BEHP-LDHs exhibited a two-times-greater peak response current than the NiAl–NO_3_-LDHs when exposed to MP because of the organophilic interactions between BEHP and MP. As 4-NP is the product of natural MP decomposition, it is essential to detect MP in the presence of structurally similar 4-NP. SWV found a 0.5–3.5 μM linear detection range of MP with minimal 4-NP interference and a 22.8 nM LOD. The hydrophobic BEHP intercalants selectively adsorb hydrophobic MP against the hydrophilic 4-NP. Organic molecules can be intercalated into LDHs to yield sensors with high selectivity for hydrophobic organic compounds against structurally similar derivatives.

Optical detection methods may not depend on the catalytic ability of MgAl-LDHs, eliminating the need to increase conductivity. Rather, the hosting ability of LDHs may be improved. LDHs have been extensively studied as an effective host material for various spectroscopic detection methods. Tian et al. fabricated an ultra-thin-film MgAl-LDH composite doped with Au and hygroscopic sodium polyacrylate (PAAS) for the spectroscopic detection of various organic molecules, including rhodamine G6, methylene blue, and Congo red [150]. The MgAl-LDH immobilized surface-enhanced Raman scattering (SERS)-active Au nanoparticles, preventing Au aggregation. Instead of conductivity, controlling the layer thickness is more crucial for LDHs hosting probe materials. Thick films prevent effective analyte diffusion, whereas thin films lack sufficient probe molecules. The layer-by-layer fabrication of the LDH/Au@PAAS bilayer film was optimized to 10 bilayers (Figure 10B), with each bilayer 16.81 nm thick. Increasing the Au@PAAS content increased the density of the Au@PAAS nanoparticles between the LDH sheets and increased the roughness of the thin film surface. While Au nanoparticles are effective SERS-active materials, suspended Au nanoparticles suffer from aggregations, limiting their analytical ability for various organic analytes [150]. Immobilizing the Au nanoparticles homogeneously in the MgAl-LDH lattice prevented Au aggregation, increasing SERS response. Rhodamine G6, methylene blue, Congo red, crystal violet, acid red, and Nile blue were all detected with low detection limits of 1 nM, 1 nM, 0.1 nM, 0.1 mM, and 0.1 nM, respectively. Distinct SERS peaks are distinguishable for thiram (Figure 10C) and malachite green (Figure 10D) for concentrations as low as 0.1 mM and 0.1 pM, respectively. In another example, Jin et al. optimized the number of Zn_2_Al-LDH/8-amino-1,3,6-naphthalenetrisulfonate (ANTS) bilayers to yield an optimized dextran-40 sensor [153]. Exfoliated Zn_2_Al-LDHs and ANTS were deposited on a quartz glass substrate via alternating dip-coating. The number of Zn_2_Al-LDH/ANTS bilayers was optimized to 20, exhibiting a maximum absorption peak at 222 nm. Each LDH/ANTS bilayer had a 2.05 nm thickness—in total a 41 nm-thick, thin-film coating. Interestingly, 40 ZnAl_2_-LDH/ANTs bilayers nearly exhibited fluorescence intensity, while 5, 10, and 15 bilayers showed significantly lower intensities. This suggested that 20 bilayers exposed the maximum amount of ANTs for reaction with dextran-40, and the balance between dextran-40 diffusion and accessible ANTs was balanced even beyond 20 layers. The addition of dextran-40 reduced the fluorescence of ANTS, owing to the formation of dextran-40/ANTS complexes. A linear detection range based on the diminished fluorescence was 0.1–100 mM, with an LOD of 2.7 μM. The minimum number of bilayers for maximum optical response is important to determine how much the amount of materials used needs be reduced for more light-weight and affordable sensors.

Successfully intercalating large probe molecules for optical detection often involves an ion exchange step that requires improvement. Fujimura et al. fabricated a thin-film MgAl-LDH with pyrene-1-sulfonate anions for fluorometric toluene detection [154]. The MgAl–CO_3_-LDH underwent ion exchange for replacement with propionate anions, second ion exchange with acetate, and final ion exchange with pyrene-1-sulfonate (Pyr). The lamellar MgAl–Pyr-LDH exhibited incomplete CO_3_^2−^ de-intercalation despite the multiple ion exchange steps, owing to CO_3_^2−^ having a high affinity for the positively charged brucite-like layers. If possible, fewer ion exchange steps are recommended for higher yields. Furthermore, urea hydrolysis should be avoided, and the synthesis should occur under a nitrogen atmosphere to reduce the initial CO_3_^2−^ content. Exposure to higher concentrations of toluene decreased the fluorescence intensity due to the toluene intercalation displacing Pyr. The change in fluorescent intensity could be used to determine toluene concentrations. Thus, a higher initial Pyr content without partial CO_3_^2−^ intercalation would have increased the detection range. For similar optical detection mechanisms, CO_3_^2−^ generating synthesis methods should be avoided.

LDHs as hosts for spectroscopically active probe molecules are also more sensitive to the pH of their environment. As sensor applications for organic molecules may require use in a wide range of pHs, it is essential to discuss the effects of pH on LDH stability. Zhou et al. used a MgAl-LDH to stabilize photoluminescent europium complexes (Eu) for the fluorometric determination of tetracycline [53]. Figure 10E illustrates the synthesis process and structure of the composite. Briefly, the MgAl-LDHs synthesized via a hydrothermal process underwent silanization and was subsequently reacted with Eu(NO_3_)·6H_2_O in a bicarbonate buffer solution, yielding a MgAl-LDH/Eu composite. The MgAl-LDH/Eu exhibited a disc-like, lamellar structure with an average size of 100 nm. The LDHs immobilized Eu, enhancing the thermal stability of Eu. While LDHs retained their structure in a wide pH range between 5 and 11, acidic environments dissolved the hydroxide precipitates. Thus, LDHs are practical for alkaline environments. However, a high pH of 12 resulted in Eu leaching. For different intercalants, the pH threshold for leaching may be different and must be accounted for. Increasing the tetracycline concentration increased the fluorescence intensity in a linear range between 0.1 and 10 μM, owing to increased tetracycline-Eu chelation. The LDH/Eu fluorophore exhibited a low LOD of 7.6 nM and high selectivity against common amino acids, metal cations, and polyatomic anions. Within the stable pH range, the MgAl-LDH/Eu probe exhibited excellent stability with little change to crystallinity and peak fluorescence after two months. LDHs provide a stable structure within more alkaline environments, which must be accounted for in organic molecule detection.

**Figure 11:**
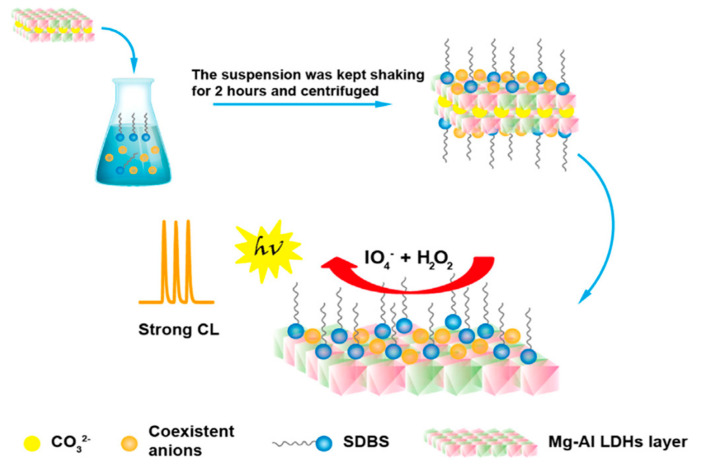
Schematic of the synthesis, structure, and CL catalysis of MgAl-LDH, reproduced from reference [155] with permission from Elsevier B.V., 2014.

Bimetallic LDHs with M^2+^ and M^3+^ metal centers have the benefit of tuning the net positive surface charge by adjusting the ratios of the metals. Increasing the ratio of trivalent metals, such as Al^3+^, leads to a more positive surface charge, which is useful for adsorbing negatively charged analytes. Guan et al. modified the Mg^2+^ and Al^3+^ ratios in MgAl-LDHs that catalyzed the CL reaction between sodium dodecylbenzene sulfonate (SDBS) detection [155]. MgAl-LDHs with different metal ratios were first synthesized via the co-precipitation of Mg and Al hydroxides at different loadings. Figure 11 illustrates the structure and catalytic role of the MgAl-LDH with a CL IO_4_-H_2_O_2_ system catalyzed by the adsorbed SDBS. The MgAl-LDH with an Mg:Al ratio of 2:1 exhibited a higher CL intensity than an Mg:Al ratio of 4:1, owing to the increase in positive surface charge density with a higher Al^3+^ content. The more positive brucite-like layers exhibited enhanced anionic exchange for SDBS instead of CO_3_^2−^. The optimized MgAl-LDH probe exhibited a linear detection range of 0.1–10 μM with a low 0.08 μM detection limit for SDBS. The standard addition of SDBS to river water resulted in excellent recoveries between 97% and 103.3%. MgAl-LDHs can adsorb and catalyze CL systems for effective optical detection. Conclusively, the ratio of M^2+^ and M^3+^ metal centers can be easily adjusted to be more positive for adsorbing negatively charged analytes and reversed for neutral or more positively charged compounds.

Beyond surface charge, the initial metal salt loadings play a significant role in LDH structure. For organic optical sensors, large nanostructures with a high surface area are desirable. Zhang et al. found that a minimal Al(NO_3_)_3_·9H_2_O loading was required to yield structurally stable ZnAl-LDHs as stable hosts for SERS-active 4-mercaptobenzoic acid [156]. The ZnAl-LDHs were synthesized via a hydrothermal process with Zn(NO_3_)_2_·6H_2_O and varying Al(NO_3_)_3_·9H_2_O concentrations. Low concentrations of Al(NO_3_)_3_·9H_2_O yielded a nanorod structure, owing to the formation of Al-doped ZnO until at least 25 vol% of Al(NO_3_)_3_·9H_2_O was added, wherein the desired plate-like ZnAl-LDH was formed. The ZnAl-LDH comprised the typical hexagonal lamellar plate structure with a thickness of 6 nm and a high specific surface area of 52.1 m^2^ g^−1^. The large ZnAl-LDHs adsorbed 4-mercaptobenzoic acid via hydrogen bonds and diffusion. The SERS signal of 4-mercaptobenzoic acid was enhanced when combined with the ZnAl-LDH compared to the Al-doped ZnO, owing to the increased adsorption and electron transfer afforded by the porous ZnAl-LDH structure. Unlike metal oxide hosts that suffer from instability, the doped ZnAl-LDH exhibited long-term stability, with no significant SERS intensity loss after 60 days. It is crucial to react the proper ratio of metal salts to yield structurally stable LDHs.

As LDHs are deposited on various substrates for optical and electrochemical detection, the morphology of the substrate may significantly influence LDH performance. Murai et al. synthesized nano-sized ZnAl-LDH on a plasmonic lattice [157]. The co-precipitation reaction of the appropriate metal chloride salts with acetylacetone and ethanol yielded nano-ZnAl-LDHs, which exhibited a smaller 70 nm diameter than the typical micrometer-length ZnAl-LDHs with NaOH precipitation. A thin film of the nano-ZnAl-LDH was deposited onto a periodic lattice of Al cylinders via spin-coating. Fluorescent fluorescein was adsorbed into the LDHs. The nano-ZnAl-LDH were homogeneously coated onto the Al lattice with dense packing to limit light-scattering losses. The plasmonic Al lattice enhanced the fluorescence intensity of fluorescein by 18 times compared to that without the Al cylinders. The plasmonic lattice resonated the excitation and emission lights for a visible increase in emissions intensity.

LDHs can directly catalyze the redox reactions of various organic sensor materials for electrochemical detection. Without compositing the LDHs with carbon nanostructures, the electrochemical response can be enhanced by synthesizing a high surface area structure and doping with catalytic compounds. Amini et al. synthesized ZnAl-LDHs doped with electrocatalytic NiCo_2_O_4_ for the selective, electrochemical determination of pyridoxine (Vitamin B6) in the presence of other vitamins [45]. The ZnAl-LDH and NiCo_2_O_4_ were prepared separately via hydrothermal reactions, yielding a NiCo_2_O_4_/ZnAl-LDH composite that comprised spherical nanoparticles with cauliflower-like surface morphology. The ZnAl-LDH retained its CO_3_^2−^ intercalants and incorporated the small, spherical NiCo_2_O_4_ onto its lamellar nanostructure. The addition of NiCo_2_O_4_ increased the surface area by creating more folds in the agglomerated ZnAl-LDH nanospheres. The peak oxidation current depended on proton accessibility, as indicated by the decrease in response current with an increasing pH, optimized to a pH of 7. The ZnAl-LDH/NiCo_2_O_4_ composite exhibited a large peak oxidation current when exposed to Vitamin B6 due to the electrocatalytic activity of the LDHs and NiCo_2_O_4_. DPV determined a wide linear response range of 0.2–200 μM and a low 86 nM detection limit for Vitamin B6. The LDH-based sensor demonstrated excellent selectivity against common biomolecules, such as caffeine, and other vitamins, such as Vitamin B2, owing to the selectivity of NiCo_2_O_4_. The probe also exhibited good stability and replicability, as indicated by 95.2% current retention after 15 days, 2.7% RSD for 5 consecutive cycles, and a 3.8% RSD for 5 identical probes. A standard addition assay of Vitamin B6 in human blood samples found high recoveries between 96.4% and 106.2%. ZnAl-LDHs are promising materials as electrochemical sensors for organic compounds owing to their high catalytic activity and ability to host other catalytic compounds.

While the importance of the surface area and porosity for effective LDH-based gas sensors has been discussed (see Section 4.4), other factors such as the film thickness and operating temperature must be considered. Xiao et al. fabricated a Zn_2_Al-LDH/1,3,6,8-pyrenetetrasulfonate (PyrTS)/ZnS thin-film electrochemical ethanol gas sensor [158]. Quartz glass was alternatingly dip-coated with a Zn_2_Al-LDH suspension and PyrTS solution to yield a Zn_2_Al-LDH/PyrTS LDH. The modified electrode underwent a sulfurization reaction with H_2_S to convert a portion of the Zn_2_Al-LDH into ZnS, yielding a Zn_2_Al-LDH/PyrTS/ZnS probe. Increasing the number of LDH/PyrTS bilayers increased the film thickness and increased the final ZnS composition. The resistance of the Zn_2_Al-LDH/PyrTS/ZnS film with 30 layers in clean air (R_a_) versus in ethanol-doped air (R_e_) was used to calibrate the sensor’s detection abilities. Increasing ethanol concentrations decreased the probe’s resistance, increasing the R_a_/R_e_ ratio. The LDH-based sensor exhibited a 50 s response time and a 16 s recovery time after ethanol gas exposure. High operating temperatures increased the adsorption of oxygen gas, resulting in faster reaction kinetics. However, temperatures above 70 °C resulted in oxygen desorption, reducing the sensor response. The sulfurization reaction increased the response current, owing to the ZnS and PyrTS having a high affinity for oxygen. ZnAl-LDHs are porous nanomaterials that can be easily modified with more adsorptive compounds for electrochemical gas sensing. Moreover, the operating temperature can be controlled for enhanced sensitivity.

Methods of improving LDH-based sensors for organic molecules were analyzed, with emphasis on enhancing electrochemical and optical detection methods. The performance of various LDHs in detecting various organic compounds is summarized in Table 6. LDHs are promising materials for different detection methods as they may be flexibly used as excellent electrochemical catalysts and/or hosts for other probe molecules. The adaptability of LDHs is one of the key features that enable their application to a wide range of organic molecules.

## 5. Future Prospects

Much research is focused on improving the detection of various analytes using fast, accurate, and selective probes. LDHs are excellent 2D materials for sensor applications due to their high surface area and excellent catalytic properties. However, high thickness and low conductivity are major constraints. By developing controlled synthesis processes, very thin layers of stacked LDHs can be synthesized on current collectors. Exfoliated LDHs with a single layer also exhibit excellent catalytic ability. Typically, a two-step process is followed for exfoliation. Initially, carbonate anions are exchanged with anions that exhibit less attraction for the brucite-like LDH layers. Then, high boiling and toxic polar aprotic solvents, such as formamide, are used for exfoliation. However, in recent reports, this can be achieved with low boiling and environmentally friendly co-solvents of water and isopropyl alcohol without exchanging carbonate anions.

Alternative non-toxic and low boiling solvents that do not require ion exchange need to be explored. In order to enhance LDH conductivity, LDHs undergo ion exchange, exfoliation, and stacking with rGO for superlattice stacking. Alternative methods in which low boiling solvents can be used to prepare LDH/rGO stacks without ion exchange will make the process simpler for conductive electrode fabrication.

Liquid exfoliation to generate monolayer LDHs, a gallery of a few lamellar LDHs, or stacking with conducting 2D graphene is not the only method to enhance the conductivity of transition metal hydroxides. Conducting polymers, such as PANI, incorporated with LDHs, also enhance conductivity due to the conjugated double bonds. At the same time, conductive polymers exhibit high electrochemical activity and strongly interact with the hydroxyl groups of the stacked thin layer of LDHs for increased stability. Alternative conducting polymers and electrode fabrication methods via in situ polymerization in the presence of LDHs may improve the interaction between the LDHs and polymers, owing to the intercalation of polymer chains into the LDH gallery.

Nickel foam and copper foam improve the surface area of the current collector. Further improvement in the structure of current collectors will reduce the interfacial resistance between with active LDH layer. The formation of hierarchical structures on the surface of electrodes with higher conductivity and deposition of LDHs onto it will form channels for enhanced electron flow and better contact between the analyte and electrode.

The development of LDH-coated electrodes with binary or ternary systems that will allow the simultaneous detection of more than one analyte will reduce the time and cost of electrochemical sensing. There are few reports on this, and more work needs to be conducted.

## 6. Conclusions

LDHs are promising materials for electrochemical and optical sensors, owing to their excellent catalytic properties and versatile hosting capability. The large surface area and lamellar structure of LDHs provide abundant active sites for rapid redox reactions, increasing the sensitivity of electroanalytical detection. The morphology of LDHs can be controlled via template-based synthesis for improved catalytic activity. While the poor conductivity of bare LDHs limits electrochemical response, LDHs can be easily composited with conductive metals or carbon nanomaterial for improved electrochemical performance. Moreover, LDHs can be directly synthesized on conductive substrates, such as Ni foam or carbon cloth, for better electron mobility and high sensor stability. The exfoliation of LDHs can reduce nanoparticle size and increase the surface area of LDHs for improved catalytic activity.

As a host material for spectroscopically active compounds, LDHs prevent agglomerations and inhibit non-radiative energy states for enhanced optical response and increased sensor reusability. LDHs can host fluorescent, CL, absorptive, or SERS-active compounds for optical analyte detection. LDHs can host catalytic compounds that interact with various analytes, allowing versatile detection methods. The ability to intercalate specific, organic, and inorganic anions allows LDHs to be easily modified to selectively detect analytes. LDHs are highly tunable materials for the affordable, accurate, and rapid detection of harmful substances.

## Figures and Tables

**Table 1:** The detection performance of LDH-based glucose sensors.

LDH	Synthesis	Analysis Method	Linear Detection Range (μM)	LOD (nM)	Highlights	Ref.
CoNi	Hydrothermal	Chronoamperometry	5–14,800 *	1600	Ni nanoparticles were extracted from the CoNi-LDHs to improve conductivity	[116]
CoNi	Electrodeposition	Chronoamperometry	20–7700 *	-	Direct synthesis of CoNi-LDHs on Cu(OH)_2_/GCE for higher conductivity	[48]
CoNi	Hydrothermal	Chronoamperometry	1–1500	680	Structural optimization via tuning Co-to-Ni ratio and LDH growth duration	[47]
CoNi	-	Chronoamperometry	1–2000	3100	Co-facilitated glucose oxidation, whereas Ni controlled morphology	[117]
NiFe	Urea hydrolysis	Chronoamperometry	0–3100	-	Ni-to-Fe ratio did not significantly influence LDH morphology	[118]
NiFe	Hydrothermal	Chronoamperometry	2–800	590	Fe content was crucial in yielding thinner, more uniform NiFe-LDHs	[46]
NiAl	Co-precipitation	Chronoamperometry	10–6100	1	LDHs with Au nanoparticles and CNTs/rGO improved conductivity	[119]
NiAl	Electrodeposition	Chronoamperometry	0.5–10,000	234	Pd improved OH^−^ adsorption and NrGOs increased surface area	[120]
CoAl	Hydrothermal	DPV	0–1	4	LDHs provided stability and high surface area for highly catalytic ARS-PBA	[121]
CoFe	Electrodeposition	Colorimetry	1–20	470	Simultaneous electrochemical and optical detection	[122]

* Combined multiple linear detection ranges.

**Table 2:** The detection performance of LDH-based dopamine sensors.

LDH	Synthesis	Analysis Method	Linear Detection Range (μM)	LOD (nM)	Highlights	Ref.
ZnNiAl	Hydrothermal	Chronoamperometry	0.001–1	13.5	rGOs increased oxidation peak currents, separating the DA, UA, and AA peaks	[42]
ZnAl	Co-precipitation	Chronoamperometry	7–500	170	MWCNTs	[127]
NiCo	Hydrothermal	Chronoamperometry	0.05–1080	17	Ni(OH)_2_ nanoboxes synergized with the LDHs for improved electron mobility	[128]
NiFeP	Hydrothermal	Chronoamperometry	0.01–500 *	0.57	Phosphorization of NiFe-LDHs improved electron mobility	[130]
MgAl	Solvothermal	Chemiluminesence	0.5–101	350	Vertical MgAl-LDHs had higher oxidation potential than horizontal LDHs	[131]

* Combined multiple linear detection ranges.

**Table 3:** The detection performance of LDH-based H_2_O_2_ sensors.

LDH	Synthesis	Analysis Method	Linear Detection Range (μM)	LOD (nM)	Highlights	Ref.
CoMn	Co-precipitation	Chronoamperometry	110–1200	86,000	Pure LDH crystal phase achieved by tuning the Co-to-Mn ratio	[40]
NiFe	Hydrothermal	Chronoamperometry	0.5–840	500	Direct synthesis of LDHs on Ni Foam increased conductivity and porosity	[134]
NiFe	Ion Exchange	Colorimetry	10–500	4400	Exfoliation increased surface area for increased peroxidase-like activity	[50]
CoNi	Hydrothermal	Colorimetry	10–90	760	Pt dopants were required for peroxidase-like LDH activity	[52]

**Table 4:** The detection performance of LDH-based sensors for nitrogen-based toxins.

LDH	Analyte	Analysis Method	Linear Detection Range (μM)	LOD (nM)	Highlights	Ref.
ZnTi	Ammonia gas	Resistance change	0.2–50 *	200 ^+^	Excellent host with high surface area for ammonia-reactive PANI	[136]
ZnTi	NO_2_ gas	Resistance change	0.2–10 *	50 ^+^	rGO content increased surface area and conductivity of LDHs	[138]
MgAl	Melamine	Fluorimetry	0.03–0.1	4	Immobilization of Ag–CTA nanoparticles increased fluorescent response	[51]
MgAl	Nitrite	CV	3.7–177.4	30	Carbon nanofibers provided a porous structure with uniform nucleation sites	[141]
NiFe	Nitrite	Chronoamperometry	5–1000	20	LDH nanosheets were thinner and larger by optimizing the Ni-to-Fe ratio	[142]

* Gas linear detection range units in ppm; ^+^ gas LOD units in ppb.

**Table 5:** The detection performance of LDH-based metal ion sensors.

LDH	Analyte	Analysis Method	Linear Detection Range	LOD	Highlights	Ref.
ZnCr	Hg^2+^	Extraction	0.013–500 μg L^−1^	4 ng L^−1^	Simultaneous Hg^2+^ extraction and detection with lamellar LDHs	[143]
MgAl	Hg^2+^	Fluorimetry	2.5–100 μM	0.00013 pM	Extremely low LOD using sensitive primuline dye	[144]
MgAl	Fe^3+^, Cd^2+^, Pb^2+^	Colorimetry	-	-	Multi-metal detection using same MgAl-LDHs with different anions	[145]
MgAl	Al^3+^	Fluorimetry	0.2–120 μM	23 nM	Simultaneous electrochemical and optical detection enabled by ARS guests	[57]
		CV		10.1 nM		
ZnAlNd	Cr^6+^	Fluorimetry	200–1000 ppb	8 ppb	Cr^6+^ steals light from LDHs, resulting in lower fluorescent response from LDHs	[146]

**Table 6:** The detection performance of LDH-based sensors for various organic compounds.

LDH	Analyte	Analysis Method	Linear Detection Range (μM)	LOD (nM)	Highlights	Ref.
CoAl	α-Naphthol	DPV	0.3–150 *	62	Exfoliation improved growth of ZIF-67 on LDHs	[49]
CoAl	Trichloroacetic acid	SWV	2500–410,000	820	Biocompatibility with hemoglobin	[147]
NiAl	Acetylcholine	Chronoamperometry	5–6885	1700	Negatively charged CQDs repelled cationic interferants	[148]
CuAl	Glyphosate	DPV	0.00296–1.18	1	Graphene increased LDH sensor conductivity	[149]
NiFe	Kojic acid	Chronoamperometry	1–4500 *	730	MOF-templating increased surface active sites	[151]
NiAl	Methyl parathion	DPV	0.5–3.5	22.8	Intercalated hydrophobic BEHP to selectively detect MP	[152]
ZnAl	Dextran-40	Fluorimetry	10–10,000	2700	The number of ZnAl/ANTS bilayers was optimized	[153]
MgAl	Tetracycline	Fluorimetry	0.1–5	7.6	LDHs are more stable hosts in alkaline environments	[53]
MgAl	SDBS	Chemiluminesence	0.1–10	80	Increasing M^3+^ ratio improved adsorption of anionic IO_4_^−^	[155]
ZnAl	Vitamin B6	DPV	0.2–200	86	Catalytic NiCo_2_O_4_ increased surface area and selectivity	[158]

* Combined multiple linear detection ranges.

## Data Availability

Not applicable.
